# Phase II clinical and exploratory biomarker study of dacomitinib in recurrent and/or metastatic esophageal squamous cell carcinoma

**DOI:** 10.18632/oncotarget.6056

**Published:** 2015-10-09

**Authors:** Hyo Song Kim, Sung-Moo Kim, Hyunki Kim, Kyoung-Ho Pyo, Jong-Mu Sun, Myung-Ju Ahn, Keunchil Park, Bhumsuk Keam, Nak-Jung Kwon, Hwan Jung Yun, Hoon-Gu Kim, Ik-Joo Chung, Jong Seok Lee, Kyung Hee Lee, Dae Joon Kim, Chang-Geol Lee, Jin Hur, Hyunsoo Chung, Jun Chul Park, Sung Kwan Shin, Sang Kil Lee, Hye Ryun Kim, Yong Wha Moon, Yong Chan Lee, Joo Hang Kim, Soonmyung Paik, Byoung Chul Cho

**Affiliations:** ^1^ Division of Medical Oncology, Department of Internal Medicine, Yonsei Cancer Center, Yonsei University College of Medicine, Seoul, Korea; ^2^ Yonsei Cancer Research Institute, JE-UK Laboratory of Molecular Cancer Therapeutics, Seoul, Korea; ^3^ Department of Pathology, Yonsei University College of Medicine, Seoul, Korea; ^4^ Department of Hematology-Oncology, Samsung Medical Center, Seoul, Korea; ^5^ Department of Hematology-Oncology, Seoul National University Hospital, Seoul, Korea; ^6^ Macrogen Inc., Seoul, Korea; ^7^ Division of Hemato-Oncology, Chungnam National University Hospital, Daejeon, Korea; ^8^ Division of Hematology-Oncology, Department of Internal Medicine, Gyeongnam Regional Cancer Center, Institute of Health Sciences, Gyeongsang National University School of Medicine, Jinju, Korea; ^9^ Department of Hematology-Oncology, Chonnam National University Hwasun Hospital, Hwasun, South Korea; ^10^ Department of Hematology-Oncology, Seoul National University Bundang Hospital, Seongnam, Korea; ^11^ Department of Hematology-Oncology, Yeungnam University Medical Center, Daegu, South Korea; ^12^ Department of Thoracic and Cardiovascular Surgery, Yonsei University College of Medicine, Seoul, Korea; ^13^ Department of Radiation Oncology, Yonsei University College of Medicine, Seoul, Korea; ^14^ Department of Radiology, Yonsei University College of Medicine, Seoul, Korea; ^15^ Division of Gastroenterology, Department of Internal Medicine, Yonsei University College of Medicine, Seoul, Korea; ^16^ Division of Pathology NSABP, Pittsburgh, PA, USA; ^17^ Severance Biomedical Science Institute, Yonsei University College of Medicine, Seoul, Korea

**Keywords:** epidermal growth factor receptor, tyrosine kinase inhibitor, esophageal squamous cell carcinoma, biomarker

## Abstract

The purpose of this study was to investigate the clinical activity, safety and predictive biomarkers of dacomitinib, an irreversible pan-HER inhibitor, in patients with recurrent or metastatic esophageal squamous cell carcinoma (R/M-ESCC). Patients, whose diseases were not amenable to curative treatment and had progressed on platinum-based chemotherapy, were treated with dacomitinib 45mg/day. The primary endpoint was objective response rate by RECISTv 1.1. Predictive biomarker analyses included the characterization of somatic mutations and gene expression using the Ion Torrent AmpliSeq Cancer Hotspot Panel and Nanostring nCounter, and investigation of their relationship with clinical outcomes. Of the 48 evaluable patients, 6 (12.5%) achieved partial responses and 29 (60.4%) had stable disease. The median response duration was 7.1 months. The median progression free survival (PFS) and overall survival (OS) was 3.3 months (95% CI, 2.4-4.3 months) and 6.4 months (95% CI, 4.4-8.4 months). Adverse events were mostly grade 1-2. Gene set enrichment analysis revealed that ERBB signaling pathway is significantly enriched in patients with PFS ≥4 months (*n* = 12) than PFS < 4 months (*n* = 21) (*p* < 0.001). Upregulation of ERBB signaling pathway was significantly associated with longer PFS (5.0 *vs.* 2.9 months, *P* = 0.016) and OS (10.0 *vs.* 4.8 months, *P* = 0.022). The most frequent mutations were *TP53* (61%) followed by *CDKN2A* (8%), *MLH1* (8%), *FLT3* (8%) and *EGFR* (8%). Dacomitinib demonstrated clinical efficacy with manageable toxicity in platinum-failed R/M-ESCC. Screening of ERBB pathway-related gene expression profiles may help identify patients who are most likely benefit from dacomitinib.

## INTRODUCTION

Esophageal cancer is the sixth most common cause of cancer deaths worldwide [1]. Two major histologic subtypes of esophageal cancer, adenocarcinoma and squamous cell carcinoma, have different epidemiology and risk factors. Esophageal adenocarcinoma, which is associated with gastroesophageal reflux and obesity, has become more common in Western countries. In many Asian countries, however, esophageal squamous cell carcinoma (ESCC), of which risk factors are smoking and alcohol abuse, represents the most common esophageal cancer [2].

Despite modest improvements in survival with multimodal therapy, the prognosis for patients with locally advanced ESCC remains dismal, with a 5-year survival rate below 30%. The majority of patients with locally advanced disease will develop both local and distant recurrences and will die within a year after recurrence. Platinum-based chemotherapy remains the backbone of treatment in recurrent or metastatic ESCC (R/M-ESCC). However, clinical benefit of the platinum-based chemotherapy is typically modest with reported objective response rate (ORR) of 20 to 35% and median overall survival (OS) of 7 to 9 months [3, 4]. Although a number of patients who progress after platinum-based chemotherapy may still be fit for second-line treatment, no treatments are available with proven efficacy for these patients.

Over the past decade, molecularly targeted therapies, which block important oncogenic pathways, have made remarkable progress, especially in epidermal growth factor receptor (EGFR) mutation-positive non-small cell lung cancer (NSCLC). In contrast, there have been only a few clinical trials with targeted agents in R/M-ESCC. Moreover, despite potential clinical and/or biological heterogeneity, most clinical trials included both squamous and adenocarcinoma histologies without the identified druggable target that showed clinical benefit. Given a high rate of older age and/or comorbidities, there is a pressing need for biomarker-directed targeted therapy to improve the efficacy and tolerability in ESCC patients.

The EGFR family (EGFR/ErbB1/HER1, ErbB2/HER2, ErbB3/HER3, ErbB4/HER4) plays an essential role in mediating cell proliferation, angiogenesis, and metastasis. Therefore, it has become an important therapeutic target in NSCLC, breast cancer and head and neck squamous cell carcinoma. EGFR overexpression and amplification was frequently observed in ESCC and was correlated with advanced tumor stage and poor prognosis [5]. Moreover, overexpression of HER2-4 has been reported to be present in 30% to 80% of ESCC [6, 7].

In this context, there is a strong rationale for investigation of biologic agents targeting EGFR family in ESCC. Gefitinib and erlotinib are EGFR-tyrosine kinase inhibitors (TKIs) which selectively block EGFR signaling through competitive reversible binding at intracellular EGFR-TK domain. In a small phase II study, gefitinib showed limited activity (ORR, 2.8%; OS, 164 days) in second-line treatment of advanced esophageal cancer [8]. Of note, a higher rate of disease control with gefitinib was observed in female patients with ESCC and high EGFR expression. Erlotinib also exhibited higher ORR (15% *vs*. 0%) and longer time to progression (3.3 *vs*. 1.6 months) in ESCC, compared with adenocarcinoma [9]. Based on these studies, further evaluation of EGFR-targeted therapy in advanced ESCC is strongly warranted.

Dacomitinib (PF-00299804) is a potent, irreversible pan-HER inhibitor. Dacomitinib demonstrated encouraging clinical activity against EGFR mutation-positive NSCLC and head and neck squamous cell carcinomas [10, 11]. Because EGFR family members act together *via* hetero- and homodimerization to activate oncogenic signaling pathways, combined inhibition of all EGFR family kinases may have more potent antitumor activity than the EGFR inhibition alone.

This phase II study (ClinicalTrials.gov identifier NCT01608022) assessed the efficacy, safety, and predictive biomarkers of dacomitinib in patients with R/M-ESCC who progressed after 1 or 2 chemotherapy regimens.

## RESULTS

### Patient characteristics

Between June 2012 to Aug 2013, a total of 49 patients were enrolled into the study and were received at least one dose of dacomitinib. The patient characteristics are listed in Table 1. The median age was 64 years. Majority of patients were male and ECOG PS 0-1. Approximately 40% of the patients had both locoregional and distant diseases in at least 3 organ sites. Two thirds of patients (73.5%) had received two or more treatment modalities including surgery, chemotherapy, and radiotherapy prior to enrollment. Thirty-three patients (67.3%) received dacomitinib as second-line treatment and sixteen patients (32.7%) received as third-line chemotherapy. Approximately 60% of patients received 5-fluorouracil and cisplatin prior to enrollment. The median time from initial diagnosis to the study enrollment was 10.3 months (range, 2.5 to 93 months).

**Table 1:** Baseline patient characteristics (N=49)

Characteristics	No. of patients	%
Age, years		
Median, range	64	47-83
Sex		
Male	48	98.0
Female	1	2.0
Performance status		
0	7	14.2
1	38	77.6
2	4	8.2
Smoking status		
Never-smoker	6	12.2
Former smoker	31	63.3
Current smoker	12	24.5
Smoking dosage		
Median, range	20 (0-80)	
Stage at diagnoses		
I	6	12.2
II	11	22.5
III	20	40.8
IV	12	24.5
Disease status at study entry		
Locoregional	18	36.7
Distant	13	26.5
Both	18	36.7
Previous treatment		
Chemotherapy alone	13	26.5
Surgery + CT	13	26.5
Radiation + CT	17	34.7
Surgery + RT + CT	6	12.2
No. of prior chemotherapy regimen*		
1	33	67.3
2	16	32.7
Prior chemotherapy regimen		
Cisplatin alone	2	4.1
PF regimen	38	77.6
DP regimen	23	46.9
TPF regimen	2	4.1

Abbreviations: PF, cisplatin and 5-fluorouracil; DP, docetaxel and cisplatin; TPF, docetaxel, cisplatin and fluorouracil; RT, radiotherapy; CT, chemotherapy

* Chemotherapy given as adjuvant, part of multimodality treatment, or palliative treatment

### Efficacy and treatment delivery

**Figure 1:**
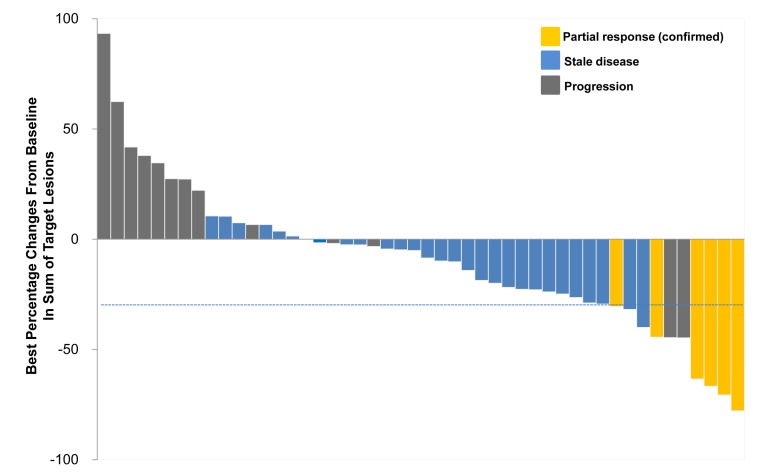
Waterfall plot of maximum percentage changes from baseline in sum of the largest diameter of target lesions (N=48)

Response was not evaluable in one patient because of rapid clinical deterioration. The waterfall plot of maximum percentage changes from baseline for 48 evaluable patients are shown in Figure 1. Of the 48 evaluable patients, 6 patients (12.5%) had confirmed partial responses (Table 2). The six patients had PRs lasting 16.6+, 8.5, 7.1, 4.1, 2.0, 2.0 months, respectively, with the median response duration of 7.1 months. Twenty-nine patients (60.4%) had stable disease and 13 patients (27.1%) experienced progression. The 8-week DCR was 72.9 % (35/48). The median number of treatment cycle was 3 (range, 1-21) and the median treatment duration is 2.9 months (range, 0.6-21.6 months) with 9 patients (18.4%) treated for longer than 6 months. Reasons for treatment discontinuation were disease progression (*n* = 43, 87.8%), refusal of the patient because of adverse events (*n* = 3, 6.1%), and other medical conditions (*n* = 2, 4.1%, infection).

**Table 2:** Best response by RECIST 1.1 (N=48)

Characteristic	No. of Patients	%
Best response		
Complete response	0	0
Partial response (confirmed)	6	12.5
Stable disease	29	60.4
Progressive disease	13	27.1
Nonevaluable*	1	NA
Objective response rate (95% CI)	12.5% (5.9 to 24.2)	

* Response was not evaluable in one patient because of rapid clinical deterioration

NA: not available

With a median follow-up of 12.1 months, the median PFS was 3.3 months (95% CI, 2.4-4.3 months). Fifteen patients (30.6%) were progression free at 4 months and were considered to achieve clinical benefit (Supplementary Figure 1A). The median OS was 6.4 months (95% CI, 4.4-8.4 months) and 5 patients (10.2%) were alive at 1 year (Supplementary Figure 1B). There was no significant difference in PFS and OS for performance status, initial stage at study entry, and body weight.

### Adverse events

All the 49 patients were assessed for treatment-related AEs (Table 3). AEs were mostly grade 1 to 2 and easily manageable. The most common AEs were diarrhea (69.4%), acneiform rash (67.3%) and mucositis (61.2%). Treatment-related grade 3 AEs occurred in 13 patients mainly acneiform rash in 5 (10.2%), mucositis in 2 (4.1%), and diarrhea in 2 (4.1%). Nineteen patients (38.8%) had treatment interruption due to toxicity and 14 (28.6%) had a dose reduction. Of those 14 patients, 11 (78.6%) required a dose reduction to 30mg daily and 3 (21.4%) required a dose reduction to 15 mg daily.

**Table 3:** Treatment-related adverse events (N=49)

Toxicity	All grades		Grade 3	
	No. of patients	%	No. of patients	%
Hematologic				
Anemia	6	12.2	0	0
Nonhematologic				
Diarrhea	34	69.4	2	4.1
Acneiform rash	33	67.3	5	10.2
Mucositis	30	61.2	2	4.1
Anorexia	24	49.0	1	2.0
Paronychia	11	23.5	0	0
Fatigue	8	16.3	0	0
Hand-foot syndrome	7	14.3	0	0
Creatinine elevation	4	8.2	1	2.0
Nausea	4	8.2	0	0
Myalgia	3	6.1	0	0
Dyspepsia	2	4.1	0	0
Dysphagia	2	4.1	0	0
Dry skin	2	4.1	0	0
Hemoptysis	1	2.0	1	2.0
AST elevation	1	2.0	1	2.0
ALT elevation	1	2.0	0	0
Vomiting	1	2.0	0	0
Arthralgia	1	2.0	0	0
Neuropathy	1	2.0	0	0
Dry eye	1	2.0	0	0

### Association of gene expressions with clinical outcomes

**Figure 2:**
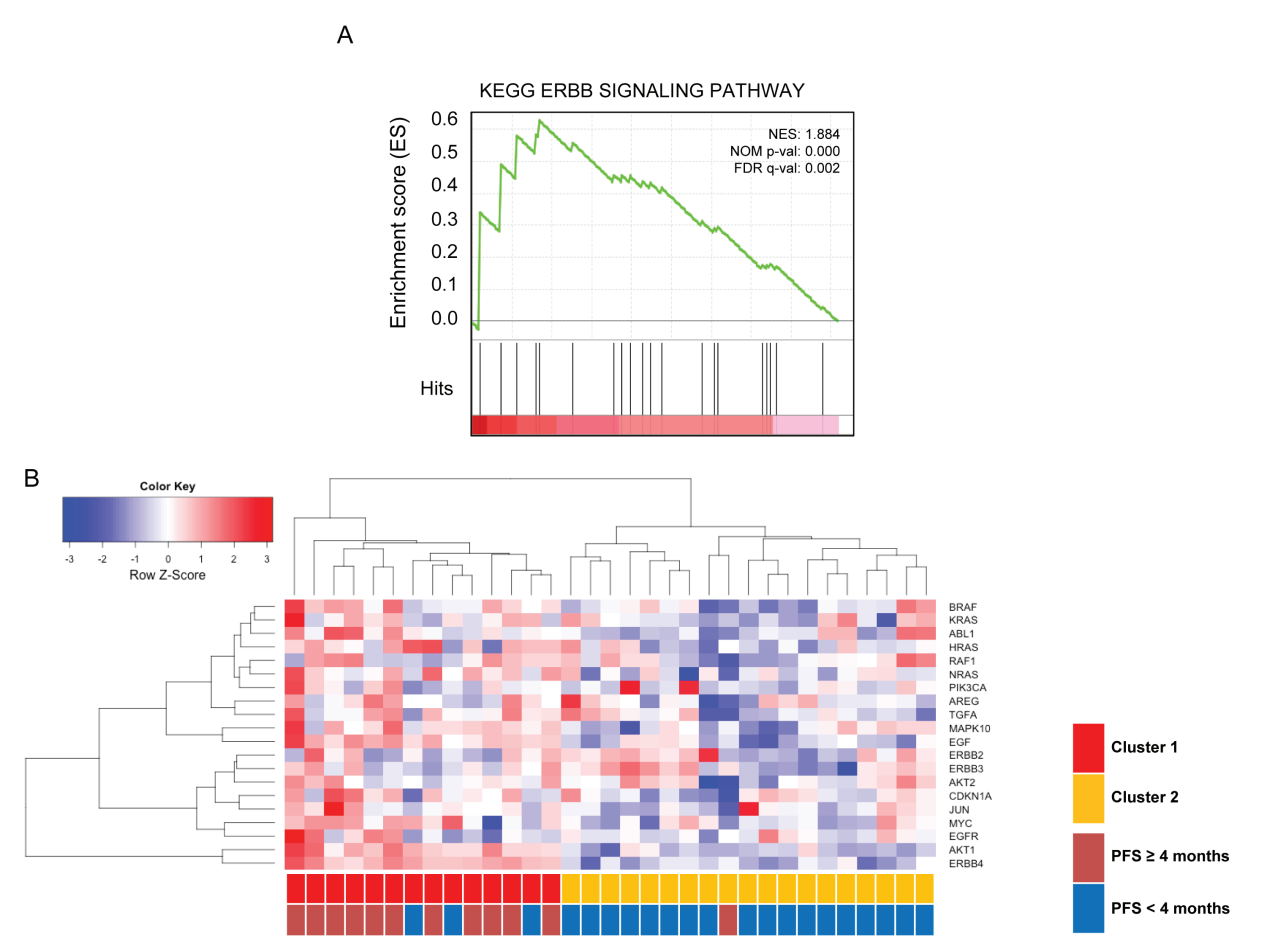
**A.** GSEA enrichment plot of KEGG ERBB pathway genes. Genes in the ERBB signaling pathway showed significant enrichment in patients with clinical benefit (PFS ≥4 months) *vs*. patients with non-CB (PFS <4 months). The top portion of the figure plots the enrichment scores (ES) for each gene, whereas the bottom portion of the plot shows the value of the ranking metric moving down the list of ranked genes. The table enumerates the genes in the ERBB pathway for which a majority of probe sets were significantly enriched and upregulated in in patients with clinical benefit *vs*. patients with non-clinical benefit. **B.** Hierarchical cluster analysis of gene expression (n= 33). Samples were represented in descending order of PFS (from left to right). The first row indicated subgroup according to gene expression profiles (cluster 1 *vs*. cluster 2). The second row indicated subgroup according to clinical benefit to dacomitinib (PFS <4 months *vs*. PFS ≥ 4 months).

Gene expression profiles were available in 33 (67.3%; Figure 2, Supplementary Table 1). The GSEA of gene expression profiles against Kyoto Encyclopedia of Genes and Genomes (KEGG) database was used to identify differentially enriched signaling pathway between patients with CB and non-CB on dacomitinib. Among the 27 upregulated human KEGG pathway gene sets, this approach identified 20 significant pathways at 25% false discovery rate (FDR) level (Supplementary Table 1). Notable among them were the ERBB signaling pathway, given the biologic and predictive potential of ERBB pathway activation. The pathway enrichment plot and expression profiles of a subset of genes contributing significantly to the core enrichment in patients with CB were shown in Figure 2A and 2B. Of the 87 pathway-affiliated genes of the ERBB pathway, 20 genes including *ERBB4, EGF, EGFR, AKT1,* and *MAPK10* were differentially expressed between patients with CB (*n* = 12) and non-CB (*n* = 21) (*P* < 0.001; Figure 2B). Hierarchical cluster analysis demonstrated 33 tumors could be classified into 2 clusters, each with distinctive expression pattern of ERBB pathway genes. All but one patient showing CB on dacomitinib belonged to cluster 1 with upregulation of ERBB pathway genes.

**Figure 3:**
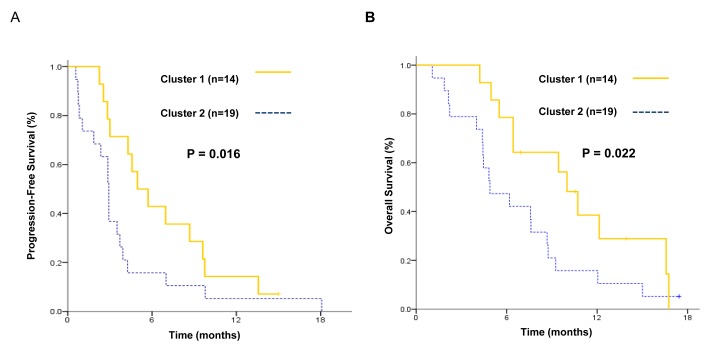
**Kaplan-Meier estimates of A.** progression-free survival and **B.** overall survival for patients with upregulated ERBB pathway (cluster 1) compared to those without (cluster 2).

Notably, patients with upregulated ERBB pathway (cluster 1, *n* = 14) showed significantly longer PFS (5.0 *vs.* 2.9 months, *P* = 0.016) and OS (10.0 *vs* 4.8 months, *P* = 0.022), compared to those without (cluster 2, *n* = 19) (Figure 3A, 3B). Furthermore, among the 32 evaluable case, patients with upregulated ERBB pathway (3 out of 14) tended to have higher ORR (21.4% *vs.* 5.6%, *P* = 0.18), compared to those without (1 out of 18).

### Association of somatic mutations with clinical outcomes

**Figure 4:**
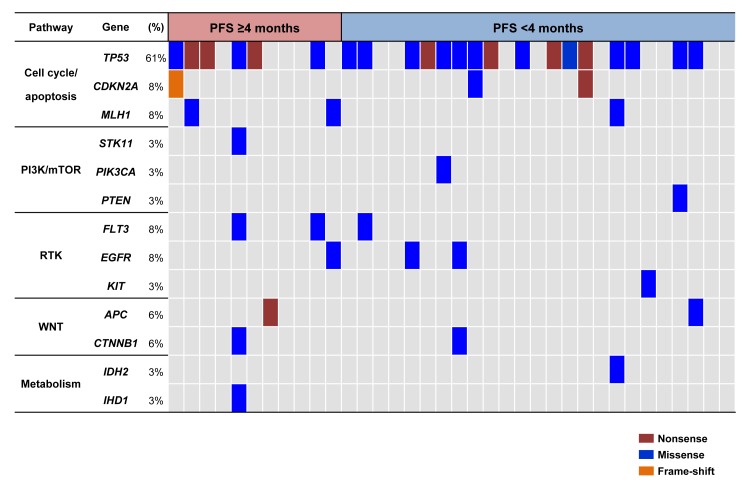
Somatic mutation profiles between patients with non-clinical benefit (PFS <4 months; n = 25) and patients with clinical benefit (PFS ≥4 months; n = 11) Samples were represented in descending order of PFS. Gene symbol, frequency of mutations and involved pathways were shown in the left panel.

Somatic mutation results were available in 36 patients (73.5%; Figure 4). Median sequencing depth in target regions was 730X. Overall, we identified 44 somatic coding single nucleotide variants (SNVs) and small insertions/deletions (indels). The full list of somatic mutations is provided in Supplementary Table 2. The list of the 13 recurrently mutated genes in two or more tumors or well-known driver cancer genes involved in 5 important oncogenic pathways (cell cycle, PI3K/mTOR, receptor tyrosine kinase, WNT and metabolism) is presented in Figure 4. The most frequently mutated genes were *TP53* (61%) followed by *CDKN2A* (8%), *MLH1* (8%), *FLT3* (8%), and *EGFR* (8%). *EGFR* mutations were found in 3 patients (1 CB, 2 non-CB). Interestingly, all these *EGFR* mutations were not classic drug-sensitive exon 19 and 21 mutations frequently identified in NSCLC, but atypical exon 20 mutations (V765M, C775Y, and G810D). No specific mutation appeared to be enriched in patients with CB.

### Association of ERBB expression and sensitivity to dacomitinib

**Figure 5:**
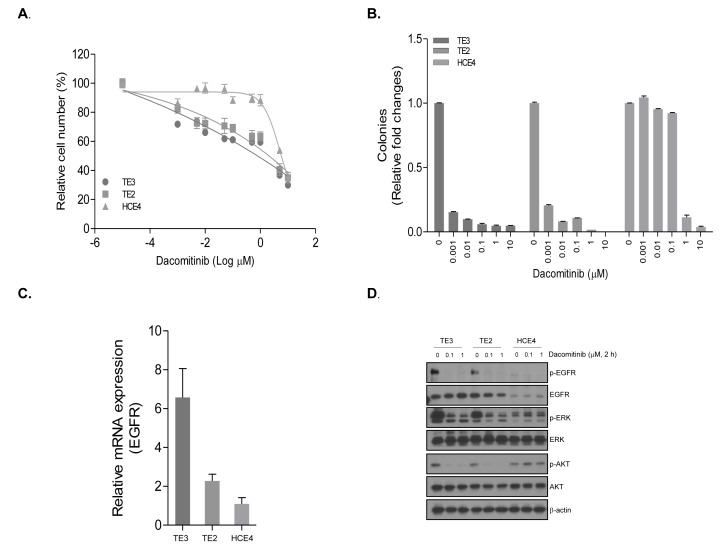
The anti-tumor efficacy of dacomitinib in ESCC cell lines with different EGFR expression The viability of TE2, TE3, and HCE4 cell lines were measured by proliferation **A.** and clonogenic assay **B.**. The relative cell viability (%) and colonies represents the percent growth as compared to the control group (no treatment). **C.** Expression of EGFR was analyzed by quantitative real-time PCR. Expression of each EGFR mRNA is presented as relative to the mRNA expression of the internal control gene β-actin. **D.** The protein levels were measured by Western blot after 2 h treatment of dacomitinib.

To evaluate the association of drug sensitivity with ERBB gene expression, we performed detail analysis of cell viability assay with dacomitinib. Three ESCC cell lines with various levels of EGFR were treated with dacomitinib. Significant inhibition of tumor cell growth by dacomitinib was found in cells with high-EGFR expression (TE2 and TE3) in comparison to that against low-EGFR expressing HCE4 (Figure 5A–5C). The clonogenicity was deceased in TE2 and TE3 cell lines, but not in HCE4. To determine dacomitinib mediated down-regulation, we assessed phosphorylation in the EGFR signaling pathway. Dacomitinib dramatically reduced phosphorylated EGFR and AKT, and ERK in TE3 and TE2 ESCC cell lines whereas no change was detected in HCE4 cell line (Figure 5D). Taken together, high EGFR expression was associated with sensitivity to dacomitinib in ESCC cell lines.

**Table 4:** Significant KEGG gene set enriched in patients with clinical benefit to dacomitinib

	Gene set	Size	ES	NES	Nominal p value	FDR q value	Rank at Max
1	KEGG_JAK_STAT_SIGNLING_PATHWAY	19	0.71	2.07	0.000	0.000	37
2	KEGG_APOPTOSIS	17	0.68	2.04	0.000	0.000	24
3	KEGG_NEUROTROPHIN_PATHWAY	19	0.67	1.99	0.000	0.000	19
4	KEGG_ENDOCYTOSIS	17	0.75	1.90	0.000	0.002	41
5	KEGG_CYTOKINE_CYTOKINE_RECEPTOR_INTERACTION	30	0.65	1.90	0.001	0.002	41
6	KEGG_ERBB_SIGNALING_PATHWAY	20	0.63	1.88	0.000	0.002	42
7	KEGG_T-CELL_RECEPTOR_SIGNLING_PATHWAY	15	0.61	1.87	0.000	0.002	33
8	KEGG_REGULATION_OF_ACTIN_CYTOSKELETON	20	0.59	1.83	0.001	0.003	40
9	KEGG_PATHWAYS_IN_CANCER	85	0.49	1.81	0.000	0.004	57
10	KEGG_MELANOMA	29	0.52	1.78	0.000	0.005	49
11	KEGG_PROSTATE_CANCER	31	0.50	1.78	0.000	0.005	69
12	KEGG_GLIOMA	25	0.51	1.78	0.000	0.004	49
13	KEGG_CELL_CYCLE	28	0.51	1.76	0.002	0.006	69
14	KEGG_ACUTE _MYELOID_LEUKEMIA	18	0.51	1.70	0.005	0.010	26
15	KEGG_PANCREATIC_CANCER	25	0.49	1.68	0.001	0.011	49
16	KEGG_P53 _SIGNALING_PATHWAY	20	0.51	1.67	0.001	0.012	77
17	KEGG_MAPK_SIGNALING_PATHWAY	36	0.52	1.67	0.005	0.012	42
18	KEGG_NON_SMALL_CELL_LUNG_CANCER	20	0.51	1.63	0.008	0.019	49
19	KEGG_FOCAL_ADHESION	31	0.50	1.60	0.037	0.023	62
20	KEGG_ENDOMETRIAL_CANCER	19	0.50	1.59	0.012	0.024	40

KEGG, Kyoto Encyclopedia of Genes and Genomes; ES, Enrichment score; NES, Normalized enrichment score

## DISCUSSION

Because the majority of R/M-ESCC patients will not benefit from second-line chemotherapy but will nevertheless be subjected to potentially life-threatening toxicities,[12] the development of effective targeted therapy with predictive biomarker is of vital importance in this population with poor prognosis. Nevertheless, therapeutic advances in R/M-ESCC significantly lag behind those for other solid tumors, such as NSCLC [13, 14].

To the best of our knowledge, this is the first report on efficacy, safety and predictive biomarkers of pan-HER inhibitor in esophageal cancer. In our study, dacomitinib showed promising clinical activity with manageable toxicity in heavily-pretreated R/M-ESCC. We performed next-generation sequencing and nanostring nCounter gene expression assay in R/M-ESCC patients treated with dacomitinib to identify potential predictive biomarkers to this experimental agent. Using this approach, we demonstrated that the upregulation of ERBB signaling pathway may characterize a subgroup of patients who are most likely to benefit from dacomitinib.

It seemed apparent that the clinical benefit of the selective EGFR-TKI (gefitinib, erlotinib) or the EGFR-specific MoAB (cetuximab) were limited in esophageal cancer, with reported ORR of 2.0%-6.6% and PFS of 1.6-1.8 months [8, 9, 15–18]. However, the efficacy of dacomitinib in our study (ORR, 12.5%; PFS, 3.3 months) exceeded reports from previous studies with selective EGFR-targeted therapies. Furthermore, we observed 8-week DCR of approximately 72.9% in this population with poor prognosis, which could partly be attributed to the potential of dacomitinib to simultaneously block compensatory signaling from other HER receptors. The pan-HER inhibitory effects of dacomitinib might induce disease stabilization rather than tumor shrinkage, resulting in modest ORR but high DCR. Our results deserves attention, given that patients enrolled in our study had multiple poor prognostic factors (one third had progressive disease after 2 prior chemotherapy regimens and had both locoregional and distant diseases).

Treatment with dacomitinib in heavily pretreated R/M-ESCC patients was well tolerated with the frequency of AEs comparable to that seen in other studies with EGFR-targeted agents. All toxicities reported in our study were mostly grade 1-2 and easily manageable with supportive care. There has been controversy regarding the relationship between acneiform skin rash and clinical outcome to EGFR-targeted therapies [19, 20]. However, acneiform skin rash were not found to be associated with clinical outcomes in our study.

The EGFR-targeted agents have demonstrated only marginal benefit in unselected patients [8, 9, 15–17]. Therefore, selection of patients who are most likely to respond to EGFR-targeted agents including dacomitinib is an important challenge. Conflicting data exist regarding the correlation between EGFR expression and response to EGFR-targeted agent [8, 9, 21]. Furthermore, conclusions that patients with EGFR overexpression derived benefit from gefitinib or erlotinib were made based on small sample size, which hampers the validity of such analysis. In our study, comprehensive biomarker analysis identified that the screening for ERBB-related gene expression could help identify subgroups most likely benefit from dacomitinib in R/M-ESCC. Several studies have shown that gene expression signatures could serve as useful molecular markers complementary to genetic mutations or protein expressions in predicting clinical outcomes to targeted therapies. For examples, in addition to *PIK3CA* mutations, gene expression signature involved in the PI3K pathway activity may have utility in the identification of breast cancer patients likely to benefit from a selective PI3K inhibitor therapy [22]. From a study in NSCLC, mRNA expression of fibroblast growth factor receptor 1 (FGFR1) was the better biomarker of FGFR-TKI sensitivity than FGFR1 gene copy number or protein expression [23]. Very recently, we demonstrated that overexpression of inflammatory genes predicted significantly worse survival to dacomitinib in head and neck squamous cell carcinoma [11]. Collectively, identification of ERBB pathway gene expression as a potential biomarker can contribute to the successful development of EGFR-targeted therapy in R/M-ESCC. Our results need further validation in the future clinical trials with EGFR inhibitors in ESCC patients.

Recently, whole exome sequencing studies in ESCC have been reported, [24, 25] uncovering recurrent somatic mutations in *TP53* (60-93%), *CDKN2A* (3-8%), *RB1* (8-9%), *PIK3CA* (7-9%) and PTEN (5%). In our study, genes involved in cell cycle and apoptosis regulation were mutated in 69% of cases by somatic alterations of *TP53* (61%), *CDKN2A* (8%) and *MLH1* (8%), which was comparable to the previous reports. Consistent with previous reports [24, 25], we also found mutations in PI3K/mTOR pathway including *PIK3CA* (3%), *PTEN* (3%) and *STK11* (3%). Although somatic mutations in the EGFR tyrosine kinase domain have been associated with dramatic response to EGFR-TKIs in NSCLC, these drug-sensitive *EGFR* mutations are rare in ESCC [8, 9, 24, 25]. In our study, three patients harbored *EGFR* exon 20 mutations (V765M, G810D, C775Y). Interestingly, a NSCLC patient with V765M *EGFR* mutations had a partial response to gefitinib [26]. Consistent with this report, a case with V765M *EGFR* mutation in our study showed PFS of 4.1 months with 10% of tumor shrinkage. The activating mutation (D820E) in exon 17 of *KIT* tyrosine kinase in a patient showing rapid progression within 28 days has been previously reported in imatinib-resistant gastrointestinal stromal tumors [27, 28].

In conclusion, dacomitinib demonstrated promising efficacy in platinum-failed R/M-ESCC. Screening of ERBB pathway-related gene expression profiles may help identify patients who are most likely to benefit from dacomitinib. The value of ERBB gene expression profiling in predicting clinical outcome to dacomitinib needs to be validated in future randomized trials.

## PATIENTS AND METHODS

### Study design

This was a multicenter, open-label phase II study of dacomitinib in patients with recurrent or metastatic ESCC (R/M-ESCC) who had progressed on platinum-based chemotherapy. The primary objective of this study was to assess objective response rate according to Response Evaluation Criteria in Solid Tumors (RECIST) criteria 1.1 [29]; secondary end points were to estimate the clinical benefit [CB, progression free survival (PFS) ≥ 4 months of dacomitinib], duration of responses, disease control rate at 8 weeks [8-week DCR; complete response (CR)+ partial response (PR)+ stable disease (SD) at 8 weeks), PFS, OS, and the tolerability of the treatment. Exploratory objectives were to evaluate whether somatic mutation or gene expression are correlated with clinical outcomes.

All patients provided signed informed consent and the study was conducted in accordance with the Helsinki Declaration. The study was approved by the institutional review board.

### Study population

Patients with pathologically confirmed R/M-ESCC not amenable to curative treatment were enrolled. Patients were at least age of 18 years, had an Eastern Cooperative Oncology Group performance status (ECOG PS) of 0 to 2, had at least one measurable lesion, had experienced treatment failure with one to two prior chemotherapy regimens, including platinum-based chemotherapy, and had a life expectancy of at least 3 months. Patients with prior EGFR inhibitors and ≥ 3 lines of palliative chemotherapy for R/M-ESCC were ineligible.

### Treatment

Patients received continuous treatment with oral dacomitinib 45 mg once daily until disease progression, death, or unacceptable adverse events (AEs). Treatment cycle was 28 days long. Drug doses withheld and/or reduced for intolerable grade 2 or grade 3/4 toxicity. A maximum of 2 dose-level reductions were permitted (30 mg then 15 mg). Dacomitinib administration could be interrupted for a maximum of 21 days.

### Study assessment

Baseline evaluations included a complete medical history, physical and radiologic examinations, complete blood count, and biochemistries. Response evaluations were defined according to RECIST 1.1 guidelines. Radiographic imaging was conducted at week 4, week 8 and every 8 weeks thereafter until disease progression or when clinically indicated. If a patient had a CR or PR, a confirmatory evaluation was performed after 4 weeks. Safety was measured by assessment of physical examination, documentation of AEs, laboratory measurements on day 1 of each cycle. AEs were graded according to the Common Terminology Criteria for Adverse Events version 4.0 [30].

### Biomarker analyses

Fresh or archival formalin-fixed paraffin embedded (FFPE) tumor tissues were collected at baseline for biomarker analysis, which included characterization of gene expression by Nanostring nCounter^®^ Cancer Human Cancer Reference Kit, which is a sensitive assay quantifying mRNA transcripts of 230 genes using multiplexed, color-coded probes, and somatic mutations by Ion Torrent AmpliSeq Cancer Hotspot Panel v2 (CHPv2), which is a next generation sequencing assay detecting 2,800 Catalogue Of Somatic Mutations In Cancer (COSMIC) mutations from 50 genes. The sets of genes for expression and mutation analysis were listed in Supplementary Table 3. These two analytical platforms have been previously validated with FFPE clinical samples. [31–33] Analysis and normalization of the raw Nanostring data was conducted using nSolver Analysis Software v1.1 (Nanostring Technologies). For a significance analysis of gene expression, gene set enrichment analysis (GSEA; http://www.broad.mit.edu/gsea) were performed with a ratio-of-classes metric for gene ranking and 1000 data permutations. Among the 49 cases, 7 cases had insufficient tumor and 6 and 9 cases had insufficient DNA and RNA, respectively. Overall 36 and 33 cases had successful AmpliSeq and Nanostring analyses.

### Statistical rationale for study design and statistical analysis

A Fleming's one-stage design was used to test the null hypothesis (P0) with 5% significance level that the ORR is ≤ 5% *versus* the alternative hypothesis (P1) that the ORR is ≥ 15%. Forty-four response-evaluable patients were required to provide 80% power to reject P0 when the true ORR is 15%. Allowing for 10 % loss to follow-up rate, it is anticipated that the total sample size is 49. PFS was defined from the first day of dacomitinib until the first disease progression or death from any cause. OS was defined from the first day of dacomitinib to death from any cause. For the predictive biomarker analysis, we defined clinical benefit (CB) as PFS ≥ 4 months on dacomitinib based on the previous results that second-line therapies with either cytotoxic agent or EGFR inhibitors in R/M-ESCC have shown PFS of less than 4 months [16, 34, 35].

PFS and OS were estimated using the Kaplan-Meier method, and compared using generalized Wilcoxon test. The association of biomarkers with clinical outcomes was analyzed using a two-tailed Fisher's exact test. *P*-value ≤ 0.05 was considered significant.

### Experimental study

Proliferation assay: Cell culture and reagents ESCC cell line TE2, TE3, and HCE4 were purchased from Japanese Cell Resource Center for Biomedical Research (Sendai, Japan). All cell lines were cultured by RPMI1640 (Invitrogen, Calsbard, CA) added 5% bovine serum albumin and penicillin/streptomycin. Cells were seeded at 3,000 cells/well in 96 well plates and treated with indicated concentrations of dacomitinib and MTT reagents [3-(4,5-Dimethylthiazol-2-yl)-2,5-diphenyltetrazoliumbromide] for 72 hours. Cell proliferation was assessed according to manufacture instructions.

Clonogenic assay: Cells were seeded at 3,000 cells/well in 6 well plate and treated with indicated concentrations of dacomitinib for 10 days. Colonies were fixed with paraformaldehyde (4%) and stained crystal violet (0.05% w/v) for 30 min Western blot analysis: Cells were treated with indicated concentrations of dacomitinib and lysed in Cell lysis buffer (Cell signaling, Danvers, MA) containing protease inhibitor cocktail (Roch Diagnostics, Mannheim, Germany). Protein content was resolved by SDS-PAGE, and was transferred to nitrocellulose membranes (Millipore, Temecula, CA). p-EGFR (Y1068), EGFR, p-AKT(S473), AKT, p-ERK (T202/Y204), and ERK secondary antibodies were purchased from Cell Signaling Technology (Beverly, MA, USA) and β-actin from Santa Cruz biotechnology (Santa Cruz, CA, USA).

Quantitative real-time PCR: Total RNA was purified from cells using RNeasy mini prep kits (Qiagen). cDNA was prepared from 2 mg total RNA using the SuperScript first-strand synthesis system (Invitrogen Life Technologies, Inc.). Differential RNA levels were assessed using Taqman gene expression assay (Life Technologies). Quantitative PCR reactions were performed on a VIIA7 Real-Time PCR system and analysed using VIIA7 software (Life Technologies).

## SUPPLEMENTARY MATERIAL FIGURE AND TABLES


