# Supplementary material for: Phase II clinical and exploratory biomarker study of dacomitinib in recurrent and/or metastatic esophageal squamous cell carcinoma
Source: Oncotarget. 2015 Oct 9;6(42):44971–84. doi: 10.18632/oncotarget.6056 (PMC4792605; doi:10.18632/oncotarget.6056)
Supplement: Supplementary file 1 [file oncotarget-06-44971-s001.pdf]

Phase II clinical and exploratory biomarker study of dacomitinib in recurrent and/or metastatic esophageal squamous cell carcinoma

Supplementary Material

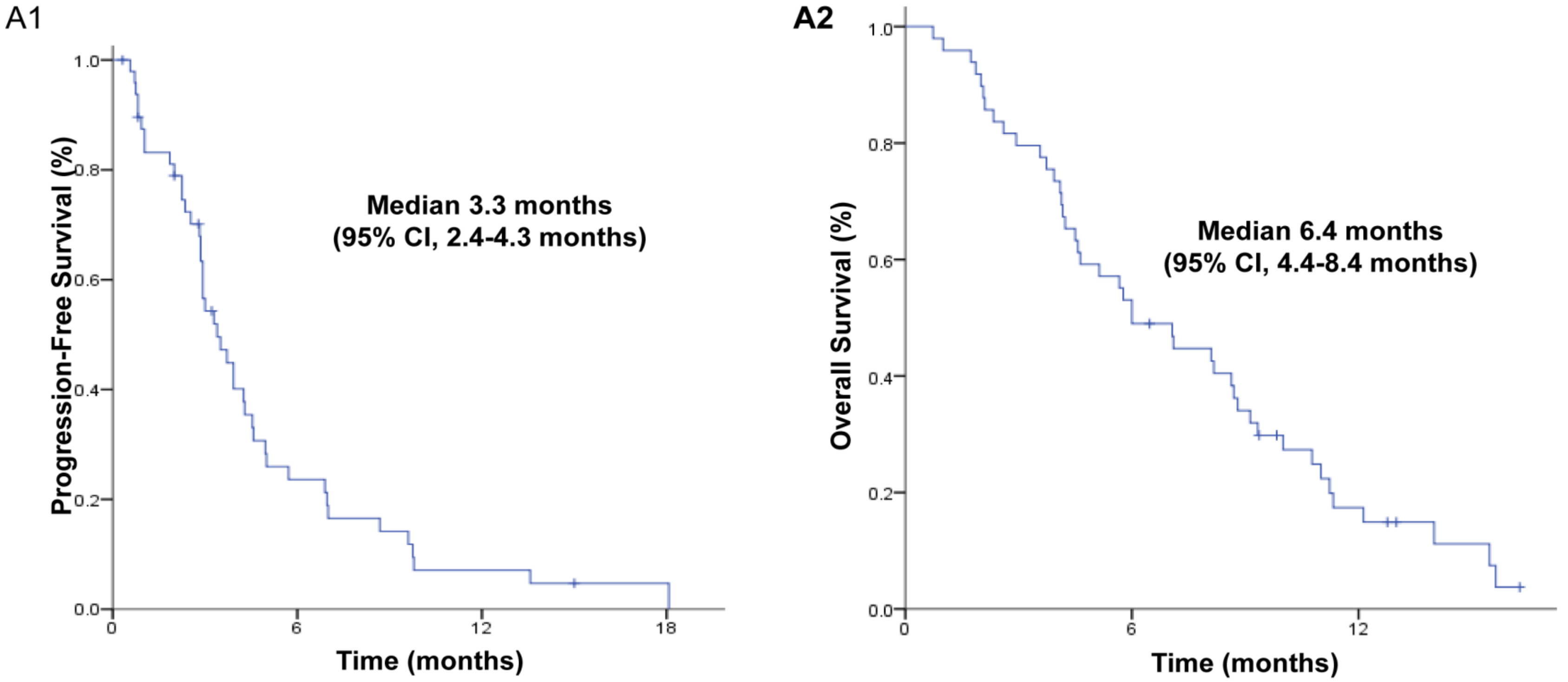

Supplementary Figure 1.

Kaplan-Meier estimates of (**A**) progression-free survival and (**B**) overall surviva

Supplementary Table 1: Gene expression profile in 230 cancer genes (n= 33)

Gene	Mean expression in CB	Mean expression in non-CB	Ratio (CB/on-CB)	*P*-value	CB 1	CB 2	CB 3	CB 4	CB 5	CB 6	CB 7	CB 8	CB 9	CB 10	CB 11	CB 12
ABCB1	165.39	34.29	4.82	0.00	710.77	76.5	47.67	76.65	16.49	172.02	305.94	104.58	222.74	12.26	81.55	157.54
ABL1	250.23	165.97	1.51	0.08	379.08	553.13	168.26	153.31	150.9	204.96	371.68	136.09	186.31	69.5	129.63	499.88
AKT1	55.60	10.47	5.31	0.00	194.8	15.69	22.43	27.38	116.49	73.2	91.02	24.35	30.18	4.09	22.15	45.44
AKT2	531.21	497.44	1.07	0.58	800.28	794.39	729.12	361.36	640.39	539.86	442.48	511.42	379.9	192.14	495.41	487.76
APC	201.87	123.59	1.63	0.03	389.61	115.73	86.93	93.08	164.85	252.54	518.33	179.07	165.49	61.32	183.36	212.07
AR	83.77	21.65	3.87	0.00	221.13	86.3	25.24	87.6	5.07	51.24	123.89	34.38	36.43	32.71	201.27	99.98
AREG	247.13	207.90	1.19	0.65	426.46	143.19	157.04	147.83	81.7	96.99	391.91	557.26	650.51	28.62	59.86	224.19
ATM	57.01	11.75	4.85	0.00	184.27	17.65	50.48	71.18	26.63	54.9	118.84	48.71	43.71	8.18	29.22	30.3
BCL2	117.05	56.03	2.09	0.00	342.22	121.61	86.93	54.75	92.57	153.72	166.88	77.36	67.65	65.41	66.46	109.06
BCL2A1	143.88	59.79	2.41	0.00	305.37	72.57	30.85	131.4	32.97	285.48	222.5	267.89	113.45	20.44	52.79	190.86
BCL2L1	625.54	493.87	1.27	0.08	937.17	778.69	745.95	782.95	456.51	938.8	745.89	554.39	584.94	102.2	363.9	515.03
BCL3	679.92	449.00	1.51	0.06	1574.23	698.28	1329.25	613.22	594.74	446.52	781.29	439.79	368.45	102.2	450.63	760.42
BCL6	441.26	390.17	1.13	0.50	215.86	737.5	675.84	191.63	972.63	269.01	295.83	371.03	230.02	339.32	160.27	836.16
BCR	448.13	374.88	1.20	0.16	721.3	404.06	661.82	350.41	484.41	294.63	553.73	530.04	267.49	298.44	371.91	439.29
BIRC2	382.23	231.56	1.65	0.02	594.94	280.49	190.69	1001.96	177.53	501.42	576.48	310.86	260.21	155.35	318.64	218.13
BIRC5	326.30	298.34	1.09	0.63	442.26	96.11	288.84	366.84	692.38	172.02	692.79	269.32	243.55	331.14	238.51	81.8
BLM	97.25	101.81	0.96	0.83	110.56	43.15	129	175.21	166.12	93.33	108.72	48.71	83.27	143.09	47.61	18.18
BMI1	115.16	96.75	1.19	0.30	200.07	125.53	89.74	71.18	92.57	208.62	176.99	91.68	86.39	49.06	81.08	109.06
BRAF	268.32	184.19	1.46	0.01	505.44	325.6	238.37	175.21	261.23	298.29	399.49	247.83	187.35	102.2	181.95	296.9
BRCA1	154.38	91.01	1.70	0.01	342.22	62.77	117.78	120.45	180.07	122.61	346.4	124.63	148.84	196.23	69.29	21.21
BRCA2	104.72	87.05	1.20	0.40	152.68	86.3	58.89	153.31	154.71	131.76	169.41	55.87	111.37	110.38	38.65	33.33
CASP10	91.44	13.84	6.61	0.01	463.32	29.42	36.46	82.13	5.07	71.37	136.54	97.41	79.1	12.26	44.78	39.38

Gene	non-CB 1	non-CB 2	non-CB 3	non-CB 4	non-CB 5	non-CB 6	non-CB 7	non-CB 8	non-CB 9	non-CB 10	non-CB 11	non-CB 12	non-CB 13	non-CB 14	non-CB 15	non-CB 16	non-CB 17	non-CB 18	non-CB 19	non-CB 20	non-CB 21
ABCB1	57.14	30.38	13.93	21.13	111.61	4.31	34.72	40.4	18.03	30.9	43.69	16.45	19.51	10.98	9.43	6.84	12.55	77.83	95.3	53.85	11.08
ABL1	111.81	160.58	100.3	150.63	426.76	107.63	136.95	277.06	148.71	267.79	194.63	78.44	99.48	105.43	77.39	108.7	132.67	438.48	180.77	116.84	64.37
AKT1	15.97	8.68	1.39	6.94	6.57	3.23	23.15	5.77	6.76	3.43	39.72	18.98	5.2	4.39	3.9	5.4	6.28	2.97	24.59	22.35	4.26
AKT2	621.12	462.2	441.62	269.57	600.75	482.19	518.88	405.32	608.38	395.96	472.68	805.94	588.43	538.12	525.12	373.63	485.87	829.43	431.02	446.01	144.09
APC	87.85	186.62	122.59	86.33	285.6	98.48	109.95	79.53	101.4	73.24	51.64	172.07	197.66	98.84	73.16	57.59	59.17	183.59	150.03	257.04	63.09
AR	11.06	15.19	26.47	13.58	45.96	19.91	13.5	23.73	6.76	26.32	43.69	11.39	3.9	15.37	25.69	9.36	8.07	54.66	52.26	21.34	6.39
AREG	45.46	1258.57	172.75	327.23	72.22	270.16	341.42	102.61	126.18	122.45	154.91	151.83	170.03	177.91	125.18	47.51	243.83	125.96	225.04	86.36	18.33
ATM	11.67	13.02	11.14	4.83	6.57	9.15	11.57	3.85	11.27	21.74	19.86	8.86	8.13	21.96	9.1	4.32	8.07	12.48	25.82	20.32	2.98
BCL2	38.7	23.87	47.37	25.66	65.66	12.92	48.22	48.74	47.32	75.53	59.58	69.59	22.76	70.28	59.83	14.04	38.55	155.67	81.78	116.84	53.71
BCL2A1	87.24	71.61	71.05	117.13	42.68	37.13	108.02	86.58	27.04	97.27	59.58	10.12	15.28	6.59	53	30.24	39.44	76.05	41.81	74.17	103.59
BCL2L1	460.15	911.38	639.44	422.32	817.41	494.03	740.71	333.5	450.65	387.95	329.69	547.84	476.27	362.4	495.21	327.91	558.48	587.61	349.25	436.87	242.14
BCL3	300.42	1291.12	936.18	227.61	712.36	304.6	380	264.87	148.71	403.97	325.72	752.8	584.53	481.01	375.88	232.17	252.8	512.15	334.49	490.71	116.81
BCL6	338.51	535.98	334.35	287.38	623.72	388.55	225.68	440.6	630.91	361.63	174.77	447.88	475.94	524.94	176.56	352.03	320.93	709.41	221.97	336.28	285.63
BCR	240.83	631.46	402.61	293.42	433.32	333.12	260.41	288.6	374.04	187.68	289.97	638.93	470.09	399.74	406.12	186.09	358.58	484.82	526.94	505.95	159.86
BIRC2	268.47	195.3	195.04	130.71	272.47	166.83	341.42	215.49	187.02	242.61	150.94	255.57	172.3	136.18	190.21	158.74	303	345.2	331.42	488.68	115.1
BIRC5	264.79	368.89	167.17	253.87	193.68	230.87	225.68	127.63	128.44	437.16	500.49	530.12	517.56	511.76	284.18	286.16	268.04	140.22	330.8	294.63	202.92
BLM	68.81	78.12	61.3	45.28	68.94	181.36	46.29	55.8	74.36	98.42	266.13	163.21	124.84	224.03	96.57	47.51	84.27	46.94	86.08	160.52	59.26
BMI1	237.76	58.59	66.87	57.05	121.46	71.04	121.52	76.96	99.14	67.52	59.58	105.01	65.67	63.7	114.13	76.31	69.03	188.34	119.9	129.03	63.09
BRAF	258.64	132.37	188.07	99.01	324.99	163.06	106.09	193.04	216.31	163.65	146.97	213.82	168.4	186.69	180.13	146.5	126.4	381.44	196.76	186.94	88.67
BRCA1	115.5	56.42	62.69	82.41	55.81	79.11	54.01	52.59	108.16	54.93	119.16	101.22	113.46	153.75	96.57	54.35	90.54	89.12	149.41	161.54	60.54
BRCA2	76.79	56.42	75.23	24.15	52.52	89.87	36.65	64.13	101.4	69.81	99.3	120.19	90.7	325.07	53.33	56.51	67.23	62.39	111.91	130.04	64.37
CASP10	22.73	8.68	8.36	6.04	22.98	1.08	38.58	7.7	4.51	16.02	31.78	1.27	2.6	13.18	4.23	4.32	1.79	12.48	28.9	45.72	7.67

CASP2	234.78	213.12	1.10	0.33	347.49	174.57	297.26	213.53	320.83	247.05	212.39	233.5	193.59	212.58	213.06	151.48	248.2	234.35	179.71	117.73	292.17	181.9	138.88	160.98	153.22	274.66	178.75	370.71	262.03	230.62	233.46	214.53	144.33	235.28	184.46	275.33	164.13
CAV1	301.26	382.12	0.79	0.46	121.09	598.24	129	394.21	98.91	168.36	429.83	415.44	442.35	261.64	162.15	393.84	267.25	611.93	172.75	1582.1	659.84	339.04	723.35	553.47	159.98	101.85	131.08	129.05	132.64	160.34	120.63	213.45	554.9	769.42	332.03	204.21	105.3
CCNA2	261.82	188.79	1.39	0.06	521.23	74.53	235.56	246.38	262.5	148.23	508.22	193.39	353.88	318.88	151.78	127.24	336.67	154.07	193.64	138.86	111.61	180.82	146.6	101.33	90.13	152.21	238.33	208.76	204.81	318.48	176.88	133.9	157.77	147.35	344.94	189.99	237.45
CCND1	1051.04	1176.35	0.89	0.69	1205.68	1057.22	1940.59	2699.28	1311.21	265.35	1067	352.41	833.7	490.58	632.1	757.39	1182.64	2104.85	1681.49	820.79	942.15	477.35	688.63	185.99	1336.18	307.84	4413.04	1724.48	2067.62	979.59	1163.72	203.01	415.05	1227.51	323.42	1383.74	1074.29
CCND2	338.88	244.00	1.39	0.26	537.03	702.2	134.61	87.6	44.38	492.27	270.54	355.27	138.43	134.91	766.44	402.93	114.89	156.24	844.23	117.13	525.24	69.96	289.34	547.7	9.01	428.01	75.47	59.46	72.17	13.18	223.38	324.67	198.11	453.33	386.75	161.54	54.14
CCND3	318.41	166.86	1.91	0.02	926.64	272.64	333.71	229.96	103.98	243.39	786.34	193.39	302.88	77.68	150.37	199.95	103.21	214.83	135.13	140.67	351.26	274.46	327.92	121.85	49.57	244.9	274.08	122.73	48.44	191.09	96.57	73.79	141.64	197.85	142.04	156.46	95.49
CCNE1	91.04	50.58	1.80	0.06	352.75	49.04	86.93	49.28	36.77	144.57	101.14	77.36	112.41	4.09	50.91	27.27	94	82.46	26.47	35.62	49.24	37.13	44.37	35.91	24.79	29.75	95.33	51.87	45.84	37.34	42.59	43.91	33.17	30.9	81.16	96.52	43.91
CD34	252.63	128.62	1.96	0.05	594.94	698.28	162.65	98.55	43.12	232.41	439.95	130.36	146.76	24.53	141.88	318.11	122.26	117.18	79.41	107.47	498.98	36.06	98.38	166.11	18.03	171.66	99.3	43.02	76.4	41.73	49.1	85.67	86.06	480.66	164.79	117.85	40.93
CD44	1748.72	1367.41	1.28	0.17	1347.84	1441.66	1348.88	2228.41	1198.35	1780.61	3924.13	2504.09	938.82	1381.8	1378.28	1511.76	474.28	2914.24	1416.8	1347.85	2055.01	1301.27	1265.38	2451.19	689.5	1222.22	1382.3	866.67	1716.84	1126.75	528.7	787.21	2984.26	1081.35	1449.87	1288.24	365.77
CDC2	305.57	246.98	1.24	0.27	547.56	96.11	325.3	427.07	158.51	172.02	450.06	201.99	416.33	437.43	358.71	75.74	724.94	158.41	186.68	169.95	131.31	146.92	277.77	205.87	218.57	155.64	365.44	387.15	251.95	336.05	229.23	199.05	141.64	139.62	258.25	328.16	173.93
CDC25B	241.07	204.00	1.18	0.34	389.61	162.8	300.06	208.06	183.87	279.99	467.76	170.47	315.37	126.73	230.5	57.56	212.57	412.29	78.01	249.04	154.29	113.01	260.41	125.06	99.14	178.53	202.58	160.68	376.46	430.49	139.82	189.69	228.59	150.91	190.61	247.9	83.98
CDC25C	68.85	20.64	3.34	0.01	305.37	31.38	61.7	49.28	27.9	51.24	108.72	48.71	40.59	49.06	40.07	12.12	30.1	6.51	6.97	10.87	3.28	8.07	15.43	12.83	13.52	25.18	63.55	44.28	15.28	30.75	8.78	6.12	10.76	15.45	27.05	65.02	13.64
CDH1	1085.36	1287.32	0.84	0.41	647.59	486.44	1620.9	985.54	2004.85	243.39	108.72	2012.72	1278.13	1283.69	1391.95	960.37	1164.82	2601.77	1785.98	815.96	679.53	2680.58	503.45	847.21	2607.02	583.65	909.62	1442.34	1432.38	1232.18	2139.5	881.88	887.48	931.62	1294.3	875.76	736.66
CDH11	278.62	248.36	1.12	0.72	563.35	537.44	185.09	175.21	62.14	719.2	321.11	93.12	258.12	102.2	138.58	187.83	125.94	143.22	71.05	255.38	298.73	224.41	648.12	666.35	22.53	892.63	162.86	59.46	71.52	52.71	87.79	522.65	323.62	196.07	226.89	127	36.66
CDK2	85.07	76.77	1.11	0.54	100.03	137.3	129	60.23	181.34	42.09	85.97	37.25	68.69	73.59	44.78	60.59	66.97	67.27	50.15	68.22	85.35	62.43	55.94	38.48	45.07	77.82	127.11	131.58	60.14	140.57	131.69	48.23	87.85	77.83	55.95	95.5	37.94
CDK4	169.96	166.36	1.02	0.85	184.27	119.65	145.82	208.06	194.02	164.7	192.16	107.44	181.1	310.7	143.77	87.86	218.71	121.52	73.84	123.77	157.57	141.54	131.17	125.06	227.58	171.66	262.16	165.74	146.94	219.64	212.97	143.26	111.16	142.6	208.44	200.15	188
CDK6	441.04	295.37	1.49	0.12	573.88	313.83	277.63	629.65	1118.46	279.99	720.6	295.1	636.98	220.76	101.34	124.21	133.93	438.33	154.64	297.64	272.47	338.5	271.98	182.14	261.38	256.35	174.77	341.61	236.67	911.5	199.64	143.62	895.55	245.38	225.04	151.38	70.34
CDKN1A	782.79	424.27	1.85	0.02	1068.79	2216.43	308.48	739.15	242.21	426.39	864.73	905.37	559.96	69.5	532.17	1460.25	138.23	1191.3	295.34	788.18	485.85	441.29	549.74	360.43	164.49	267.79	115.19	366.91	518.21	318.48	260.77	507.53	688.47	664.85	278.54	370.83	137.27
CDKN2A	42.08	17.62	2.39	0.06	179.01	39.23	16.83	60.23	8.88	40.26	35.4	54.44	14.57	12.26	7.54	36.35	2.46	17.36	20.9	5.43	9.85	2.15	13.5	7.7	4.51	22.89	47.67	5.06	49.74	112.02	1.63	5.04	7.17	8.91	14.76	9.14	2.13
CDKN2B	185.19	157.27	1.18	0.71	537.03	139.26	190.69	54.75	11.41	120.78	111.25	611.7	41.63	32.71	49.97	321.13	13.52	644.48	763.43	36.53	137.88	157.14	27	20.52	13.52	49.21	47.67	187.25	363.13	338.24	26.66	29.16	33.17	203.2	180.16	16.26	14.49
CDKN2C	56.67	55.00	1.03	0.87	15.79	100.03	109.37	54.75	58.33	69.54	40.45	48.71	51	24.53	34.88	72.71	45.46	26.04	68.26	32.3	95.2	27.45	55.94	17.32	29.29	56.08	67.53	93.63	41.61	96.64	99.82	55.43	65.44	83.18	31.97	20.32	46.04
CEBPA	269.43	215.69	1.25	0.31	494.91	178.49	277.63	164.26	434.96	525.21	182.05	362.43	98.88	85.85	307.33	121.18	487.8	225.67	179.71	53.43	151.01	123.24	131.17	128.27	529.52	141.91	87.39	411.19	336.8	259.17	140.79	168.46	106.68	164.58	152.49	430.77	119.37
CHEK1	142.77	122.85	1.16	0.26	168.48	105.92	126.19	125.93	175	96.99	283.18	138.96	150.92	143.09	131.98	66.65	149.9	95.48	100.3	51.32	128.03	106.02	94.52	88.5	96.89	151.06	218.47	216.35	112.16	129.59	106.32	76.67	172.12	70.7	164.17	139.19	112.12
COL1A1	8973.59	10070.24	0.89	0.76	14457.7	18072.8	10291.9	5650.41	1872.97	7751.95	29104.8	1173.25	12126.6	2571.46	4294.16	315.08	672.72	11029.86	720.24	22152.76	19959.2	14740.8	29649.6	15567.2	263.63	19758.1	5068.44	1869.98	1211.64	889.54	4924.76	27437.1	25190.1	1037.38	6588.34	2516.55	227.22
CSF1R	66.05	21.51	3.07	0.00	215.86	100.03	64.5	65.7	7.61	91.5	88.5	44.41	29.14	8.18	28.75	48.47	14.13	23.87	13.93	20.23	59.09	3.23	54.01	20.52	6.76	48.06	43.69	6.33	4.23	6.59	4.55	16.56	16.14	23.77	19.68	33.53	12.79
CSF3	168.88	39.17	4.31	0.02	452.79	39.23	92.54	54.75	128.08	64.05	829.33	87.39	111.37	24.53	27.34	115.12	22.12	80.29	2.79	44.37	9.85	44.13	23.15	13.47	2.25	69.81	166.83	3.8	4.55	17.57	83.24	6.84	21.51	52.88	36.28	114.8	2.13

CSF3R	150.62	108.98	1.38	0.18	142.15	72.57	53.28	104.03	178.8	391.62	169.41	283.64	130.1	16.35	44.31	221.16	218.71	212.66	210.36	137.95	59.09	44.67	82.94	160.33	227.58	180.82	47.67	39.22	39.66	21.96	85.19	40.67	101.3	146.75	78.09	73.15	79.72
CSK	322.87	303.53	1.06	0.70	631.8	202.03	381.39	202.58	327.17	428.22	323.64	335.22	242.51	155.35	244.64	399.9	207.04	433.99	509.88	140.67	433.32	373.48	310.56	197.53	356.01	226.59	154.91	538.98	395.97	144.96	236.71	178.89	202.6	409.37	311.12	524.24	87.39
CTGF	1178.83	790.96	1.49	0.41	731.83	6925.87	695.47	711.78	220.65	1202.32	806.57	189.1	473.57	81.76	192.32	1914.69	94.61	451.35	132.35	415.37	1411.59	612.43	723.35	1768.17	42.81	2752.29	762.65	48.08	391.74	105.43	405.79	2155.02	1134	2066.44	488.82	441.94	205.91
CTNNB1	1259.86	1326.28	0.95	0.65	1216.21	1979.1	1116.12	804.86	1353.05	1571.98	1016.43	1515.63	1240.66	482.4	1158.62	1663.24	1285.85	1206.49	1228.73	1217.44	1250.73	1397.07	1643.45	1801.52	1279.85	1638.79	1811.29	517.47	1354.03	579.85	908.48	1288.98	1109.8	1787.19	1937.46	865.6	1741.89
CXCL9	361.59	252.17	1.43	0.54	489.64	251.07	117.78	109.5	199.09	1967.27	308.47	87.39	494.39	20.44	224.37	69.68	126.56	19.53	110.06	175.69	160.86	44.13	2222.13	262.31	58.58	184.25	95.33	43.02	48.76	48.32	348.56	65.15	393.54	174.08	94.08	189.99	430.57
CYP1A1	65.20	8.59	7.59	0.00	300.1	33.34	22.43	65.7	2.54	82.35	83.44	61.6	62.45	4.09	40.07	24.24	16.59	21.7	2.79	1.21	9.85	1.08	15.43	2.57	2.25	13.73	11.92	1.27	0.98	2.2	1.95	0.72	0.9	1.78	31.36	39.62	0.43
DAP3	270.69	306.34	0.88	0.24	152.68	376.6	333.71	301.14	375.36	314.76	103.67	203.42	337.23	151.26	310.63	287.81	366.77	253.88	220.11	197.73	315.15	410.08	266.19	241.14	358.27	328.44	214.5	417.52	359.88	270.16	327.75	271.04	196.32	326.19	308.67	465.31	318.02
DAPK1	64.90	43.06	1.51	0.16	100.03	145.15	28.04	71.18	40.58	166.53	65.74	24.35	30.18	20.44	25.93	60.59	25.19	17.36	32.04	75.17	160.86	17.22	61.73	35.91	65.34	50.35	31.78	26.57	13.33	4.39	13.98	24.12	38.55	125.96	41.81	23.37	19.18
DEK	582.28	575.71	1.01	0.92	594.94	545.28	574.89	667.98	370.28	534.36	619.47	481.33	888.86	392.46	735.81	581.68	814.64	737.78	376.14	580.2	718.93	416	453.3	559.25	678.23	720.97	802.37	570.61	468.14	349.23	337.51	230.37	242.94	620.88	722.47	1021.05	668.87
DLC1	82.52	37.25	2.22	0.03	300.1	145.15	47.67	21.9	12.68	122.61	141.59	35.81	42.67	4.09	52.32	63.62	27.65	15.19	34.83	34.72	118.18	17.76	28.93	35.27	2.25	37.77	47.67	16.45	14.95	15.37	25.36	27.36	45.72	127.15	55.34	35.56	18.76
E2F1	101.81	109.43	0.93	0.75	194.8	54.92	126.19	120.45	204.16	54.9	121.36	64.46	91.59	89.94	56.56	42.41	92.77	75.95	50.15	82.71	75.5	59.2	86.8	54.51	87.88	99.56	353.52	203.7	182.38	169.12	122.58	70.55	91.44	56.44	96.53	79.25	107.43
E2F3	261.78	116.11	2.25	0.01	795.01	131.42	297.26	229.96	126.81	256.2	599.24	207.72	187.35	61.32	103.7	145.42	55.91	88.97	32.04	55.85	98.48	78.03	75.23	68.62	65.34	255.2	381.32	135.38	92.65	248.19	124.21	34.92	65.44	65.36	109.45	237.74	69.91
EGF	137.84	32.53	4.24	0.00	531.76	66.69	78.52	125.93	120.29	95.16	174.46	84.52	118.65	28.62	53.74	175.74	58.36	21.7	16.72	3.92	45.96	8.07	23.15	29.5	47.32	42.34	63.55	65.79	17.23	68.09	18.86	5.4	11.65	7.72	73.17	42.67	11.94
EGFR	1824.22	662.74	2.75	0.07	10235.2	364.83	398.21	580.37	2974.95	150.06	1982.29	690.49	2568.75	650.02	371.44	924.09	389.5	818.07	504.31	2551.11	558.07	488.11	705.99	418.15	730.05	369.64	242.3	732.56	1356.63	430.49	500.08	508.25	984.3	756.35	461.15	224.53	188
EGR1	373.00	202.39	1.84	0.20	221.13	2124.25	378.58	120.45	252.35	75.03	255.37	170.47	151.96	32.71	99.93	593.8	38.09	117.18	41.79	101.73	285.6	372.41	109.95	67.98	54.08	271.22	150.94	315.04	410.92	272.35	218.18	245.85	119.23	352.33	145.72	468.36	91.23
EPS8	315.18	381.99	0.83	0.33	589.68	460.94	173.87	104.03	253.62	375.15	396.96	204.85	329.94	237.11	225.79	430.2	267.86	167.09	167.17	231.84	594.18	297.6	219.9	806.16	684.99	244.9	242.3	556.69	195.38	656.72	257.85	263.48	225.9	534.14	633.93	586.21	187.57
ERBB2	499.29	599.46	0.83	0.59	231.66	476.63	804.84	186.16	1728.41	170.19	199.75	766.41	163.41	224.85	227.2	811.93	605.14	629.29	810.79	239.69	505.55	524.17	142.74	259.1	757.09	200.27	476.66	924.87	382.32	524.94	791.75	190.41	216.04	940.53	498.05	347.46	2621.78
ERBB3	348.10	330.79	1.05	0.81	410.67	227.53	625.36	125.93	620.1	183	240.2	398.25	155.08	355.67	329.49	505.94	630.33	321.15	611.58	125.58	344.69	367.56	102.23	128.27	610.63	49.21	186.69	910.95	372.56	406.33	326.13	183.57	137.16	468.78	283.46	214.37	165.41
ERBB4	69.60	9.94	7.01	0.00	300.1	29.42	30.85	32.85	106.34	54.9	103.67	70.19	41.63	8.18	35.82	21.21	3.69	4.34	6.97	1.21	9.85	0.54	7.72	4.49	6.76	13.73	59.58	1.27	1.3	6.59	0.65	2.16	1.79	2.97	35.66	33.53	3.84
ERCC2	67.55	81.68	0.83	0.23	68.44	47.07	58.89	65.7	162.32	65.88	35.4	71.63	51	69.5	48.08	66.65	97.07	69.44	87.77	60.68	72.22	86.64	61.73	59.64	94.64	28.61	87.39	185.99	79	112.02	63.73	69.83	64.54	91.5	47.35	68.07	127.47
ERCC4	119.92	60.09	2.00	0.00	321.16	66.69	78.52	98.55	109.06	150.06	204.8	118.9	79.1	69.5	66.93	75.74	65.12	71.61	43.19	42.26	75.5	33.37	42.44	57.08	78.86	35.48	79.44	74.65	46.49	79.07	64.06	59.03	35.86	71.89	75.01	99.56	31.97
ESR1	122.63	24.14	5.08	0.01	605.47	33.34	28.04	43.8	6.34	133.59	278.13	100.28	80.14	16.35	76.36	69.68	36.86	6.51	33.43	8.75	32.83	5.38	32.79	10.26	11.27	25.18	31.78	10.12	5.2	13.18	9.43	5.76	9.86	36.84	54.11	79.25	48.17
ETS1	324.05	303.75	1.07	0.78	184.27	715.93	434.67	317.56	191.48	620.38	518.33	91.68	328.9	12.26	148.95	324.16	217.48	384.08	195.04	419	502.26	226.03	837.15	443.16	54.08	355.91	254.22	127.79	161.9	90.05	340.43	260.6	489.46	492.55	207.21	167.63	152.62
ETS2	1197.80	897.26	1.33	0.11	1547.91	2034.02	2355.63	1352.38	1117.19	400.77	1067	896.77	756.68	155.35	941.79	1748.06	903.11	1594.91	727.21	1365.96	1231.04	351.96	744.57	772.17	488.96	848	452.82	1252.56	1430.76	524.94	382.05	701.18	1362.6	1506.76	545.39	1254.72	400.73
ETV1	47.74	39.81	1.20	0.52	36.85	86.3	56.09	109.5	12.68	65.88	42.98	21.49	27.06	28.62	12.73	72.71	9.22	30.38	25.08	13.58	49.24	132.39	42.44	51.31	36.05	54.93	103.28	6.33	17.56	10.98	4.88	35.63	27.79	70.7	92.23	10.16	11.94
ETV6	403.09	364.84	1.10	0.61	758.16	398.17	358.95	180.68	308.15	519.72	417.19	224.91	276.86	224.85	624.09	545.32	382.75	290.77	278.62	250.85	505.55	198.04	300.91	424.57	171.25	480.65	627.6	289.73	272.11	129.59	1133.81	153.7	226.8	439.67	240.41	366.76	497.5

FANCG	89.44	81.24	1.10	0.64	115.83	45.11	103.76	49.28	95.11	45.75	283.18	42.98	133.23	49.06	43.37	66.65	76.79	78.12	51.55	79.39	105.05	62.43	106.09	52.59	103.65	53.79	123.14	97.42	59.49	72.48	176.23	61.19	85.16	59.41	94.08	64.01	43.91
FAS	51.82	29.95	1.73	0.05	168.48	94.15	25.24	43.8	16.49	84.18	63.21	32.95	31.22	4.09	15.56	42.41	27.03	36.89	22.29	28.98	49.24	14.53	57.87	31.43	9.01	56.08	43.69	21.51	7.8	17.57	22.44	21.6	35.86	30.3	33.2	43.69	17.9
FAT1	937.39	843.23	1.11	0.47	747.63	1053.3	897.38	1023.86	895.27	708.22	1362.83	1184.71	1071.01	1222.36	503.42	578.65	475.51	969.97	448.58	751.96	856.8	798.09	582.54	692	950.87	496.67	758.68	556.69	629.39	1265.12	689.32	599.68	2167.6	1362.38	821.47	684.76	1149.75
FGF1	93.50	30.15	3.10	0.00	310.63	54.92	30.85	38.33	96.38	113.46	222.5	87.39	61.41	4.09	56.56	45.44	17.2	19.53	8.36	16	32.83	11.84	17.36	25.65	18.03	26.32	67.53	6.33	10.4	6.59	30.56	28.44	10.76	35.05	36.89	81.28	126.19
FGF2	85.32	69.68	1.22	0.63	52.65	421.71	44.87	49.28	21.56	21.96	60.68	24.35	21.86	4.09	16.03	284.78	30.1	88.97	26.47	37.13	170.7	44.13	63.65	126.34	63.09	51.5	91.36	5.06	35.44	26.36	62.1	50.75	43.93	234.69	124.2	18.29	69.06
FGFR1	236.90	270.74	0.87	0.76	458.05	729.66	148.63	136.88	51.99	204.96	225.03	104.58	128.02	69.5	109.83	475.64	68.19	101.99	48.76	152.75	581.05	122.16	144.67	329.01	252.36	130.46	1294.92	24.04	90.38	19.77	32.84	174.22	140.74	652.97	244.1	74.17	1006.08
FGFR2	132.84	227.70	0.58	0.16	63.18	198.11	193.5	114.98	257.42	27.45	48.04	124.63	75.98	73.59	274.81	142.39	119.19	121.52	117.02	38.04	206.81	287.38	82.94	475.23	968.9	438.31	27.8	191.05	260.73	133.98	57.23	115.18	94.13	281.03	322.19	55.88	387.09
FGFR3	1998.63	1246.54	1.60	0.11	1416.28	613.93	5653.51	2069.63	3705.37	186.66	1206.06	2071.46	462.13	551.9	4516.65	1529.93	1491.05	1948.62	2488.11	562.69	1267.15	751.27	412.79	481.64	1575.03	1958.07	472.68	2883.41	3769.84	845.61	668.19	622.35	1164.48	1058.18	819.01	902.18	34.96
FGFR4	117.62	21.81	5.39	0.00	442.26	94.15	84.13	104.03	3.8	133.59	235.14	57.3	53.08	106.29	46.19	51.5	16.59	15.19	4.18	12.68	29.54	9.15	36.65	6.41	22.53	62.94	79.44	3.8	24.06	6.59	5.2	7.2	15.24	11.29	41.2	42.67	5.54
FGR	249.62	90.21	2.77	0.00	721.3	147.11	67.3	191.63	46.92	525.21	530.97	179.07	179.02	36.79	85.32	284.78	147.45	134.54	79.41	107.47	164.14	23.68	185.18	126.34	90.13	201.42	71.5	25.3	25.36	10.98	32.84	47.87	53.79	91.5	91.62	91.44	92.51
FLT1	146.73	56.59	2.59	0.00	458.05	158.88	112.17	147.83	44.38	146.4	298.36	93.12	107.2	12.26	66.93	115.12	41.78	110.67	71.05	35.62	65.66	19.37	52.08	29.5	13.52	122.45	127.11	48.08	54.62	63.7	30.24	20.16	36.75	68.33	73.78	76.2	27.71
FLT3	44.12	9.20	4.80	0.00	168.48	31.38	30.85	65.7	12.68	47.58	50.57	31.52	41.63	4.09	14.61	30.3	7.99	2.17	6.97	4.23	16.41	2.15	11.57	9.62	4.51	22.89	15.89	5.06	2.6	2.2	7.8	5.4	14.34	15.45	20.91	11.18	3.84
FOLR1	213.42	35.73	5.97	0.01	952.96	41.19	39.26	71.18	11.41	226.92	604.3	150.42	206.08	8.18	103.7	145.42	69.42	21.7	13.93	8.45	82.07	9.69	25.08	24.37	13.52	18.31	31.78	32.9	9.1	8.79	16.58	6.48	9.86	99.22	105.14	140.2	3.84
FOS	2406.61	1345.83	1.79	0.23	1068.79	13475.1	3309.1	1122.42	1521.71	294.63	568.9	1396.73	808.72	73.59	383.22	4856.4	401.18	898.36	367.78	1495.16	4668.09	1686.06	709.85	644.54	504.73	1738.35	464.74	450.41	2525.04	322.87	776.14	2365.23	1491.68	2351.04	737.23	1491.44	2172.46
FOSL2	1826.40	1486.48	1.23	0.23	2206.03	3254.04	3233.38	969.11	2083.48	1127.29	1726.92	1189.01	822.25	568.26	1237.81	3499.16	600.23	1944.28	1688.46	1098.2	2357.02	1351.32	954.82	1341.04	1687.69	2312.84	929.48	1708.03	1859.56	2332.57	1152.67	1199.35	1559.81	2532.25	1029.91	1019.01	557.61
FRZB	45.53	34.57	1.32	0.49	15.79	194.18	61.7	21.9	12.68	84.18	12.64	18.62	11.45	16.35	15.08	81.8	50.38	19.53	29.26	29.58	141.16	8.07	30.86	78.24	2.25	29.75	35.75	11.39	16.58	26.36	5.85	19.08	33.17	110.51	28.28	10.16	9.81
FYN	142.85	144.20	0.99	0.97	142.15	390.33	151.43	54.75	152.17	232.41	219.97	14.33	138.43	16.35	65.52	136.33	114.27	232.18	75.23	193.5	275.75	134.54	241.12	160.33	24.79	242.61	139.02	54.4	78.02	96.64	137.21	118.78	229.49	244.19	70.71	71.12	94.21
GADD45A	379.50	311.68	1.22	0.31	758.16	845.38	134.61	158.78	369.01	362.34	283.18	435.49	253.96	53.15	366.72	533.2	143.76	468.71	189.46	214.33	361.1	435.91	162.03	214.85	162.23	183.1	79.44	416.25	562.42	371.19	447.73	417.18	365.75	436.7	231.19	466.33	215.71
GAS1	150.04	93.45	1.61	0.11	384.34	231.45	95.35	202.58	48.19	142.74	323.64	67.33	150.92	4.09	43.84	106.04	12.29	43.4	30.65	150.33	114.9	145.3	150.46	98.77	11.27	331.88	143	12.65	14.95	8.79	49.75	207.33	155.98	114.67	87.31	68.07	10.66
GATA1	15.79	4.44	3.56	0.00	47.38	11.77	16.83	16.43	5.07	10.98	45.51	8.6	10.41	4.09	9.43	3.03	3.69	2.17	2.79	1.51	3.28	0.54	3.86	5.13	6.76	10.3	11.92	1.27	3.25	4.39	2.28	1.08	2.69	7.72	7.38	9.14	2.13
GNAS	96.27	14.79	6.51	0.00	326.43	56.88	39.26	109.5	2.54	91.5	316.05	48.71	46.84	8.18	45.72	63.62	23.96	19.53	6.97	4.83	19.7	1.61	11.57	8.34	6.76	30.9	47.67	3.8	24.06	6.59	2.28	2.88	2.69	7.13	30.13	43.69	5.54
GRB7	205.28	132.53	1.55	0.13	458.05	123.57	364.56	82.13	637.85	106.14	219.97	141.82	59.33	69.5	64.11	136.33	88.47	180.11	178.32	69.43	101.77	104.94	57.87	60.29	130.69	27.47	190.66	235.33	98.18	353.62	86.17	55.43	68.13	145.57	125.43	122.93	302.25
HCK	315.78	134.16	2.35	0.01	768.69	192.22	123.39	219.01	176.26	816.19	659.92	266.45	287.27	8.18	126.33	145.42	119.19	156.24	129.56	104.75	118.18	228.72	252.69	152	198.29	230.03	107.25	50.61	115.73	68.09	86.49	91.79	135.36	131.9	126.05	141.22	73.32
HDAC1	1050.23	843.44	1.25	0.10	1189.89	751.23	1794.76	881.51	1752.5	607.57	859.67	1088.73	773.33	1111.98	1140.24	651.36	1380.46	1071.96	873.49	587.44	672.97	938.55	432.08	429.05	957.63	711.82	687.18	1423.36	707.41	1240.96	1153.64	1033.41	688.47	1040.35	815.32	546.59	320.16
HIF1A	1501.24	1276.62	1.18	0.38	1458.4	3440.38	1034.79	1390.7	740.57	823.51	2616.93	1825.06	1287.5	228.94	1607.84	1560.23	855.19	1861.82	1453.02	2949.88	1004.53	1250.69	1062.84	1426.33	876.52	2085.1	703.07	759.13	1867.36	560.08	767.69	1236.07	1069.46	1354.06	1159.03	1988.24	518.81
HMMR	105.01	15.61	6.73	0.00	442.26	45.11	36.46	104.03	11.41	75.03	230.09	85.95	83.27	24.53	67.41	54.53	33.18	8.68	18.11	11.47	6.57	5.38	25.08	10.26	11.27	16.02	27.8	32.9	7.48	8.79	3.25	6.48	5.38	9.51	43.04	29.46	7.67

HRAS	191.37	141.59	1.35	0.08	179.01	166.72	204.72	383.26	237.13	67.71	237.67	226.34	107.2	143.09	195.15	148.45	76.79	262.56	202	84.22	151.01	119.47	73.3	106.46	92.38	194.55	357.49	184.72	114.11	103.23	121.28	114.46	137.16	122.99	207.21	82.29	65.65
HSP90AB1	3699.47	3085.98	1.20	0.11	4691.11	4758.47	2807.12	2841.63	4113.69	2130.14	4639.68	2720.4	3760.49	2473.34	2989.42	6468.14	2840.79	2864.33	1824.99	2955.31	3161.3	3920.5	4330.44	1930.42	2913.46	2304.83	4798.34	2479.81	3041.94	4052.34	3108.13	3264.02	2458.05	3081.24	2659.32	4800.44	2015.58
IFNGR1	684.65	509.67	1.34	0.02	1310.98	590.4	737.54	711.78	584.59	993.7	591.65	711.97	597.43	322.97	575.07	487.76	840.44	635.8	487.59	553.93	443.17	305.68	661.62	623.38	337.99	683.21	385.3	397.28	690.83	538.12	378.8	355.27	438.36	731.39	315.43	490.71	408.83
IGF1	209.61	92.53	2.27	0.05	436.99	796.35	129	142.36	19.02	139.08	348.92	118.9	89.51	16.35	75.89	202.98	26.42	26.04	26.47	201.05	430.04	11.84	113.81	168.67	36.05	231.17	83.41	17.71	11.38	4.39	15.93	81.71	138.95	153.88	93.46	55.88	14.92
IGFBP2	984.07	821.68	1.20	0.64	1047.73	666.89	737.54	268.29	4231.62	462.99	197.22	528.61	387.19	1655.71	940.38	684.68	538.79	807.22	380.32	159.39	705.79	720.6	119.59	555.4	4010.79	1098.63	464.74	330.22	2075.75	1058.66	661.04	583.12	766.46	701.09	684.97	729.46	103.17
IGFBP3	1366.64	718.61	1.90	0.18	1621.62	843.42	908.6	788.43	328.44	7640.32	652.34	1830.79	961.72	122.65	410.56	290.84	797.44	614.1	443.01	560.27	334.84	964.38	966.39	1140.94	288.42	2161.78	417.07	380.83	816.97	502.97	300.12	675.62	2186.43	596.52	459.92	247.9	234.89
IGFBP6	1109.72	625.85	1.77	0.18	847.66	1498.55	1186.23	1286.67	740.57	203.13	2627.05	1007.08	576.62	1741.56	129.15	1472.37	164.03	592.4	39.01	642.98	1408.31	316.98	540.1	326.44	9.01	184.25	337.63	177.13	111.51	32.95	210.37	366.07	4955.55	1962.47	361.54	166.62	237.45
IL1A	160.60	90.56	1.77	0.13	500.17	62.77	92.54	153.31	29.17	104.31	240.2	318.02	281.02	8.18	61.75	75.74	89.7	525.13	22.29	58.26	72.22	120.55	13.5	16.03	11.27	91.55	67.53	58.2	82.57	74.68	89.09	56.87	98.61	54.66	105.76	188.97	4.26
IL1B	341.37	151.43	2.25	0.05	716.04	92.19	114.98	295.66	159.78	203.13	644.75	1300.75	389.27	16.35	99.93	63.62	210.72	590.23	64.08	196.52	22.98	107.09	42.44	38.48	15.77	338.74	321.74	16.45	131.01	41.73	159.98	60.11	131.78	26.14	185.08	463.28	15.77
IL4	30.43	5.27	5.77	0.00	105.3	21.58	16.83	43.8	1.27	14.64	83.44	27.22	10.41	8.18	11.31	21.21	2.46	4.34	4.18	1.21	9.85	2.15	5.79	2.57	2.25	9.16	19.86	1.27	1.3	2.2	0.65	1.44	0.9	0.59	15.37	22.35	0.85
IL6	70.33	64.90	1.08	0.84	26.32	162.8	39.26	38.33	21.56	20.13	96.08	28.65	93.67	12.26	8.01	296.9	12.9	65.1	6.97	59.77	45.96	55.43	32.79	44.89	15.77	80.11	135.05	5.06	24.06	101.03	41.62	27.36	43.93	149.13	324.04	70.1	21.74
IL8	2495.26	1949.26	1.28	0.50	3690.76	617.86	549.65	3597.21	268.84	1140.1	4214.9	8522.21	4372.5	28.62	507.66	2432.75	5043.88	6724.68	344.1	2545.98	203.53	1246.38	493.81	517.56	144.21	3617.46	4766.56	222.68	931.41	698.45	1349.71	1578.74	1746.27	480.07	1592.52	5638.61	1047.86
IRF1	223.15	144.29	1.55	0.09	131.62	627.66	187.89	240.91	410.86	378.81	106.19	67.33	140.51	20.44	205.05	160.57	127.17	88.97	123.99	66.11	137.88	64.58	300.91	79.53	87.88	271.22	178.75	412.46	99.81	123	159	85.31	146.12	190.72	48.57	145.28	92.93
ITGB1	1357.94	1196.32	1.14	0.44	1284.66	2963.75	1177.81	1292.15	626.44	2117.33	1608.09	1115.95	1307.27	466.05	645.3	1690.5	1236.7	1239.04	514.06	1317.06	2081.27	1087.08	1761.11	1596.29	549.79	1557.53	1330.67	1010.9	632.32	579.85	830.44	1237.15	1169.86	2307.07	996.71	1316.69	771.19
JUN	499.25	321.31	1.55	0.25	552.82	2187.01	415.04	279.24	371.55	300.12	257.9	355.27	248.76	73.59	292.25	657.42	137.62	262.56	123.99	325.42	331.56	256.7	281.62	141.09	220.82	203.7	174.77	145.5	519.18	336.05	263.37	1672.68	265.35	406.4	162.94	347.46	168.82
JUNB	1630.52	1035.20	1.58	0.09	1858.54	4707.47	3281.05	1691.84	1971.88	391.62	1264.22	928.29	574.53	547.81	367.67	1981.34	280.76	1718.6	824.73	573.25	1217.9	1229.16	572.89	411.74	326.72	1417.92	1660.35	1872.51	1185.63	2929.99	992.04	573.76	980.71	1278.01	411.96	1028.16	252.37
KDR	126.46	68.24	1.85	0.01	347.49	121.61	86.93	54.75	59.6	146.4	255.37	87.39	97.84	12.26	93.33	154.51	124.1	71.61	72.44	58.26	108.33	37.13	73.3	73.75	11.27	58.36	67.53	51.87	41.61	61.5	65.03	52.55	43.93	136.06	132.2	61.97	30.27
KIT	86.64	43.81	1.98	0.11	373.81	84.34	33.65	38.33	25.36	60.39	116.31	20.06	43.71	12.26	46.67	184.8	20.27	15.19	25.08	15.09	147.72	8.61	54.01	42.33	40.56	22.89	23.83	20.24	26.66	8.79	35.44	12.24	30.48	141.41	30.74	155.44	43.06
KRAS	209.83	175.87	1.19	0.27	521.23	188.3	134.61	114.98	136.95	188.49	242.73	252.13	195.67	130.82	148.48	263.57	151.13	279.92	175.53	147.01	256.06	225.49	106.09	230.88	128.44	308.99	135.05	164.48	73.15	151.55	158.02	106.54	113.85	224.59	247.18	200.15	109.13
L1CAM	102.24	60.57	1.69	0.23	94.77	286.37	25.24	38.33	5.07	146.4	235.14	34.38	234.19	4.09	10.84	112.09	22.12	130.2	12.54	48.9	321.71	27.45	121.52	39.76	2.25	12.59	19.86	8.86	10.4	2.2	5.85	15.48	137.16	286.97	6.15	33.53	6.39
LAMB1	217.28	255.16	0.85	0.45	84.24	513.9	185.09	273.76	162.32	312.93	189.63	120.33	187.35	155.35	186.19	236.31	220.55	180.11	87.77	347.15	321.71	248.63	235.33	360.43	78.86	553.89	492.54	64.53	299.09	94.44	93.64	277.52	381.89	477.69	265.62	208.27	68.64
LCK	145.66	46.55	3.13	0.01	552.82	129.46	106.56	49.28	44.38	290.97	225.03	100.28	90.55	16.35	93.8	48.47	74.34	32.55	39.01	31.09	82.07	8.61	109.95	44.89	40.56	70.95	55.61	22.77	13.65	10.98	48.12	21.24	42.13	74.27	55.95	68.07	30.69
LIF	109.09	36.90	2.96	0.01	389.61	170.65	44.87	87.6	13.95	86.01	189.63	157.58	73.9	12.26	46.67	36.35	25.19	34.72	9.75	12.38	32.83	33.9	36.65	17.96	11.27	77.82	150.94	6.33	45.51	6.59	32.84	25.92	13.45	26.14	53.49	101.6	19.61
LMO1	67.68	12.52	5.41	0.01	310.63	17.65	16.83	49.28	12.68	53.07	166.88	51.57	33.31	16.35	41.48	42.41	7.37	2.17	1.39	2.72	13.13	1.61	11.57	10.26	15.77	12.59	19.86	1.27	89.4	2.2	6.18	9.72	3.59	7.13	24.59	18.29	2.13
LMO2	49.98	25.66	1.95	0.00	105.3	86.3	64.5	54.75	7.61	54.9	60.68	40.11	28.1	4.09	35.82	57.56	33.18	36.89	58.51	21.13	52.52	5.92	17.36	17.96	15.77	49.21	47.67	12.65	15.6	17.57	4.55	9.36	16.14	30.9	35.05	26.42	14.49
LYN	93.85	67.80	1.38	0.02	63.18	133.38	129	131.4	55.8	133.59	40.45	98.85	121.78	57.23	85.79	75.74	115.5	93.31	94.73	42.87	68.94	44.67	63.65	67.34	76.61	86.97	63.55	39.22	94.93	129.59	24.06	33.84	58.27	76.64	46.73	58.93	43.48

MAP3K8	170.66	117.42	1.45	0.06	452.79	192.22	201.91	104.03	154.71	237.9	197.22	121.77	117.61	28.62	60.34	178.74	90.31	134.54	111.45	70.34	147.72	87.72	77.16	81.45	81.12	113.3	79.44	275.82	182.38	138.37	85.84	109.42	105.78	177.65	97.15	159.51	59.26
MAPK10	161.12	62.66	2.57	0.01	600.21	170.65	120.59	98.55	31.7	129.93	346.4	103.14	95.76	57.23	112.66	66.65	45.46	52.08	30.65	10.87	108.33	23.68	57.87	79.53	67.6	144.19	43.69	7.59	125.81	98.84	80.31	18	15.24	108.13	78.7	97.53	21.74
MCL1	1644.33	1364.03	1.21	0.31	1131.97	4495.64	1278.77	1067.67	1351.79	1754.99	1469.02	1476.95	1742.34	322.97	889	2750.85	1302.44	1818.42	1050.41	1418.49	2425.96	1757.63	1427.41	1235.21	923.83	1025.39	591.85	1289.25	2109.56	876.36	1002.77	1498.83	878.52	2235.77	1202.69	1756.6	817.23
MET	214.21	216.80	0.99	0.95	436.99	217.72	210.32	251.86	90.03	115.29	255.37	340.95	276.86	40.88	213.06	121.18	280.76	351.53	183.89	297.34	160.86	201.27	306.7	171.24	304.19	186.54	99.3	130.32	103.06	419.51	86.49	152.26	395.33	156.85	277.31	235.7	52.44
MLH1	194.60	120.22	1.62	0.00	342.22	198.11	190.69	136.88	232.06	237.9	275.6	161.88	148.84	130.82	107.47	172.69	94.61	106.33	98.91	70.34	147.72	91.49	90.66	61.57	96.89	123.6	242.3	175.86	123.21	182.3	125.18	87.11	125.5	196.07	132.2	88.39	64.37
MLL	277.32	176.26	1.57	0.02	573.88	260.87	238.37	60.23	171.19	376.98	538.56	181.93	200.88	200.32	270.09	254.48	154.2	154.07	144.88	91.77	390.65	175.44	152.39	140.45	103.65	246.05	297.91	198.64	156.05	147.16	140.47	87.83	154.19	320.25	219.51	123.95	101.89
MMP1	779.96	1097.74	0.71	0.44	1147.77	100.03	182.28	1105.99	173.73	126.27	1438.68	550.1	4109.17	85.85	242.75	96.95	736.62	2358.74	303.7	1266.35	36.11	3488.9	1001.11	514.99	18.03	1218.79	3614.64	98.69	216.19	63.7	1391.33	1127.72	1657.53	13.67	1651.54	2265.6	8.53
MMP14	432.23	465.69	0.93	0.68	242.19	839.5	636.58	448.97	238.4	545.35	654.87	220.61	514.17	237.11	396.89	212.07	370.46	475.22	246.58	897.16	311.86	474.66	700.2	681.74	196.03	991.05	655.4	312.51	343.63	246	309.55	719.54	565.66	284	466.07	388.1	144.09
MMP2	583.46	597.26	0.98	0.95	673.92	2645.99	199.11	416.12	92.57	821.68	472.82	201.99	690.07	49.06	220.13	518.06	200.28	405.78	37.61	826.82	1155.53	555.92	1537.36	1495.6	11.27	1555.24	305.85	56.93	53.32	21.96	146.64	1398.76	1124.14	747.44	499.28	398.26	8.53
MMP3	363.28	423.12	0.86	0.79	910.84	51	173.87	125.93	168.66	96.99	680.15	403.98	1540.42	8.18	178.18	21.21	60.21	930.91	47.37	54.34	16.41	971.92	73.3	112.23	11.27	178.53	2748.72	24.04	190.51	35.14	1036.59	55.43	239.35	4.16	847.91	1244.56	2.56
MMP9	429.32	305.30	1.41	0.46	642.33	105.92	241.17	2003.93	154.71	790.57	487.99	153.28	171.74	69.5	191.38	139.36	242.67	696.55	26.47	451.9	137.88	102.25	1793.9	363.64	81.12	722.12	238.33	34.16	102.08	177.91	142.09	192.21	487.67	85.56	193.07	118.87	20.89
MPL	89.04	11.16	7.98	0.00	421.2	54.92	36.46	82.13	8.88	89.67	184.58	68.76	35.39	12.26	34.88	39.38	17.2	8.68	9.75	1.81	22.98	1.08	11.57	2.57	6.76	16.02	51.64	2.53	1.95	8.79	2.93	1.08	3.59	2.38	32.59	25.4	2.98
MSH2	226.28	201.89	1.12	0.51	479.11	117.69	157.04	202.58	367.75	161.04	209.86	181.93	264.37	196.23	189.96	187.83	279.53	132.37	107.27	102.03	200.25	142.61	162.03	160.33	497.97	125.88	158.89	183.46	325.42	316.28	216.88	102.95	86.96	196.66	193.07	364.73	184.16
MSH6	201.75	149.29	1.35	0.01	347.49	141.22	252.39	202.58	213.04	153.72	245.26	140.39	227.94	212.58	169.22	115.12	158.5	110.67	91.95	69.73	151.01	77.5	131.17	148.15	162.23	203.7	246.27	154.36	186.61	206.46	175.58	124.18	79.78	155.07	167.24	181.86	153.04
MST1R	124.63	74.31	1.68	0.08	331.69	47.07	134.61	87.6	240.94	86.01	214.92	123.2	64.53	53.15	81.55	30.3	101.37	82.46	44.58	25.96	26.26	60.81	61.73	8.34	13.52	9.16	139.02	223.94	55.27	17.57	264.67	61.19	123.71	51.1	79.93	74.17	35.81
MTA1	679.87	790.70	0.86	0.55	1010.88	719.85	698.28	536.57	1459.57	464.82	503.16	418.3	771.25	670.46	511.43	393.84	436.19	687.87	452.76	491.75	781.3	538.16	416.65	382.24	3197.37	738.14	1132.06	1085.55	692.78	781.92	533.25	537.04	674.13	805.07	554.61	702.03	983.91
MUC1	231.71	256.41	0.90	0.80	121.09	278.53	457.1	87.6	149.63	461.16	106.19	58.73	81.18	49.06	160.74	769.51	491.49	297.28	888.81	117.43	551.5	164.14	163.96	57.08	78.86	108.72	83.41	1057.71	259.75	98.84	36.09	33.48	125.5	470.56	167.24	122.93	9.81
MYB	131.27	122.15	1.07	0.90	626.53	43.15	30.85	32.85	55.8	111.63	316.05	87.39	115.53	28.62	63.16	63.62	273.39	26.04	93.34	11.77	62.37	23.68	17.36	38.48	196.03	28.61	115.19	60.73	33.49	32.95	183.71	25.56	17.03	45.75	74.4	263.14	942.14
MYBL2	284.59	282.05	1.01	0.96	247.45	125.53	361.76	547.52	428.62	133.59	260.43	173.34	518.33	396.55	194.68	27.27	439.27	334.17	114.24	302.47	203.53	221.72	239.19	161.62	139.7	227.74	679.23	299.85	495.12	373.39	246.47	222.45	237.56	115.26	252.1	282.44	335.5
MYC	1314.35	1090.64	1.21	0.32	1579.5	2061.48	1365.7	1215.5	2105.03	256.2	1908.97	968.4	984.62	466.05	952.16	1908.63	400.56	1076.3	488.98	780.34	1073.46	611.89	981.82	530.39	1730.5	598.52	1843.07	1067.84	2052.99	1238.77	645.43	900.59	879.41	1222.75	644.38	2945.28	1190.25
MYCL1	135.69	74.32	1.83	0.08	584.41	70.61	72.91	82.13	93.84	93.33	184.58	131.79	64.53	24.53	107.47	118.15	148.67	69.44	68.26	25.06	65.66	60.81	36.65	35.91	164.49	70.95	51.64	101.22	188.88	41.73	76.41	91.43	35.86	73.67	78.09	52.83	23.02
MYCN	97.43	19.33	5.04	0.00	342.22	70.61	72.91	131.4	55.8	73.2	166.88	54.44	70.78	20.44	43.84	66.65	27.03	17.36	19.5	1.21	26.26	6.46	13.5	8.98	18.03	29.75	79.44	7.59	5.2	8.79	5.2	2.52	7.17	8.91	38.74	43.69	30.69
NF1	551.41	430.99	1.28	0.12	1368.9	358.94	619.75	295.66	588.39	505.08	849.55	472.74	503.76	441.52	303.56	309.02	495.79	305.96	309.27	201.05	413.63	440.22	366.5	391.22	698.51	336.45	409.13	502.29	372.56	764.34	515.37	361.39	280.59	548.4	445.17	450.07	442.93
NGFR	301.32	224.36	1.34	0.35	758.16	266.76	266.41	104.03	815.38	117.12	326.17	289.37	154.04	163.53	227.67	127.24	144.99	164.92	309.27	86.94	160.86	25.83	123.45	111.59	65.34	53.79	1052.62	335.28	149.55	377.78	230.21	273.2	129.09	229.34	111.29	142.24	433.98
NOTCH1	437.32	325.46	1.34	0.09	747.63	337.37	356.15	197.11	691.11	417.24	806.57	350.97	252.92	388.38	411.5	290.84	214.41	310.3	339.92	240.29	449.74	245.94	246.9	296.3	509.24	249.48	71.5	761.66	631.67	329.46	294.91	241.17	345.13	398.67	360.31	194.05	103.59
NPM1	119.47	35.50	3.37	0.01	421.2	51	53.28	60.23	48.19	102.48	316.05	57.3	55.16	24.53	86.73	157.54	39.32	17.36	20.9	12.68	32.83	16.14	32.79	26.29	20.28	37.77	79.44	54.4	15.6	136.18	23.09	13.68	11.65	23.77	54.11	60.96	16.2

NQO1	745.37	894.32	0.83	0.61	694.98	535.47	451.5	476.34	578.25	290.97	374.21	1189.01	1885.97	1618.92	170.16	678.62	831.23	1325.84	488.98	68.22	420.19	2098.83	59.8	484.85	684.99	885.77	258.19	790.76	935.96	3200.14	562.19	630.63	203.49	467	1100.62	3187.08	95.92
NRAS	274.56	196.52	1.40	0.00	447.52	215.76	325.3	405.17	258.69	234.24	300.88	282.21	221.7	110.38	304.98	187.83	261.72	288.6	221.51	249.65	187.12	118.93	202.54	140.45	81.12	208.28	178.75	145.5	248.37	184.5	238.34	196.53	160.46	182.4	185.08	213.35	233.62
NTRK1	81.39	15.85	5.13	0.01	373.81	29.42	36.46	82.13	7.61	65.88	149.18	55.87	64.53	8.18	49.02	54.53	13.52	15.19	11.14	6.64	22.98	5.38	19.29	6.41	11.27	21.74	67.53	2.53	4.23	10.98	8.45	1.44	6.28	10.69	38.74	46.73	1.71
NTRK2	386.06	976.34	0.40	0.16	379.08	380.52	145.82	886.98	509.77	100.65	245.26	813.69	392.39	327.05	233.33	218.13	57.75	418.8	275.84	58.86	164.14	2276.42	34.72	885.04	2431.26	2365.48	63.55	30.37	858.91	5785.3	276.05	637.11	141.64	577.51	2495.15	362.7	306.51
NTRK3	68.12	9.84	6.92	0.02	389.61	51	16.83	43.8	3.8	56.73	101.14	21.49	30.18	12.26	33	57.56	7.99	6.51	2.79	4.23	22.98	0.54	17.36	6.41	4.51	22.89	11.92	2.53	15.6	4.39	3.25	3.6	4.48	14.26	22.14	22.35	5.97
NUMA1	363.97	499.68	0.73	0.31	142.15	745.35	653.41	290.19	308.15	342.21	179.52	209.15	218.57	339.32	418.57	521.09	936.28	384.08	362.21	153.05	558.07	247.55	327.92	223.83	1491.66	322.72	591.85	1786.47	233.75	296.51	212.32	212.73	318.24	761.7	349.86	423.66	298.84
OGG1	193.41	83.61	2.31	0.00	563.35	156.92	120.59	104.03	183.87	258.03	389.38	148.98	136.35	12.26	89.56	157.54	87.85	39.06	69.66	41.96	118.18	45.21	69.44	73.11	83.37	84.69	99.3	99.95	77.37	74.68	106.98	46.07	100.4	162.2	114.37	103.63	58.4
PCNA	597.73	490.90	1.22	0.31	1037.2	413.87	804.84	733.68	593.47	256.2	1026.55	254.99	668.21	858.52	395.01	130.27	606.37	648.82	332.96	371.6	446.46	325.59	698.27	236.65	479.94	406.26	1386.27	630.07	608.26	487.6	793.7	244.77	249.21	245.38	368.31	587.23	155.18
PCTK1	690.70	682.64	1.01	0.92	895.05	515.86	706.69	470.87	999.26	549.01	1203.54	564.42	608.88	339.32	759.85	675.59	515.45	744.29	569.79	578.08	758.32	645.25	696.34	497.68	590.35	600.81	1279.03	979.27	1011.38	685.27	547.88	640.71	449.12	618.51	709.56	652.25	566.13
PDGFA	232.88	78.38	2.97	0.01	673.92	203.99	157.04	235.43	24.09	117.12	841.97	113.17	192.55	44.97	57.04	133.3	62.05	188.79	32.04	35.62	91.92	73.19	73.3	59.64	31.55	59.51	381.32	84.77	54.94	24.16	29.26	41.39	69.92	65.95	79.93	73.15	33.68
PDGFRA	354.04	304.56	1.16	0.58	616	925.8	286.04	104.03	213.04	424.56	389.38	137.52	243.55	65.41	128.21	714.98	276.46	184.45	189.46	270.17	797.71	268	352.99	379.67	189.27	772.47	389.27	137.91	95.9	32.95	199.64	292.64	376.51	818.73	237.34	118.87	15.35
PDGFRB	399.10	354.16	1.13	0.62	631.8	939.53	286.04	246.38	130.61	638.68	753.47	114.6	425.7	12.26	295.08	315.08	269.09	288.6	142.1	371.9	469.44	539.24	572.89	717.66	22.53	723.26	393.24	127.79	101.43	59.3	231.18	471.17	677.71	462.84	514.03	244.85	37.09
PIK3CA	250.98	231.54	1.08	0.77	647.59	223.6	201.91	120.45	272.64	263.52	407.08	174.77	272.7	175.79	130.57	121.18	138.23	138.88	142.1	137.65	213.38	192.12	183.25	243.07	775.12	225.45	99.3	852.75	156.05	171.32	137.86	154.78	108.47	291.73	188.77	174.75	137.27
PIM1	1055.55	856.64	1.23	0.48	1079.32	1922.22	1177.81	678.93	746.91	688.09	520.86	1419.65	700.47	98.12	401.61	3232.55	166.49	2988.02	1780.41	640.57	1802.24	936.4	530.46	525.25	310.95	558.47	345.58	850.22	285.44	1735.15	304.02	466.13	967.26	1514.48	710.18	395.21	176.49
PLA2G2A	241.28	137.49	1.75	0.16	705.51	396.21	137.41	71.18	12.68	197.64	414.66	131.79	108.25	16.35	88.62	615	23.35	6.51	13.93	576.27	374.23	2.15	115.74	377.75	29.29	145.34	23.83	1.27	14.95	4.39	5.53	287.6	377.4	351.73	64.56	46.73	44.76
PLAT	720.97	456.53	1.58	0.34	352.75	643.35	4043.83	410.64	1386.03	225.09	166.88	634.62	309.12	49.06	112.19	318.11	248.2	935.25	647.8	234.55	433.32	347.11	264.26	300.15	74.36	724.41	2017.84	189.78	157.35	1076.23	146.64	255.56	401.61	312.52	518.34	268.21	33.68
PLAUR	440.52	361.64	1.22	0.32	452.79	611.97	204.72	914.36	140.76	843.64	404.55	339.51	650.51	77.68	227.67	418.08	369.84	722.59	84.98	596.19	193.68	351.96	455.23	384.8	117.17	385.66	250.24	131.58	368.34	358.01	249.72	611.55	624.82	207.95	183.23	589.26	357.67
PLG	73.86	9.94	7.43	0.00	315.9	35.31	39.26	87.6	5.07	58.56	164.35	44.41	42.67	4.09	31.58	57.56	13.52	6.51	5.57	3.62	9.85	0.54	7.72	5.13	6.76	17.17	47.67	2.53	0.98	8.79	2.28	2.16	3.59	3.56	26.44	33.53	0.85
PML	255.46	215.85	1.18	0.60	742.36	154.95	210.32	104.03	384.23	230.58	389.38	128.93	242.51	73.59	231.91	172.69	186.76	210.49	130.95	124.07	200.25	156.6	156.24	94.92	114.92	103	111.22	301.12	194.41	395.35	231.51	114.46	252.8	193.69	103.91	1113.5	42.63
PPARG	110.32	50.11	2.20	0.01	347.49	109.84	61.7	87.6	35.51	177.51	174.46	118.9	58.29	32.71	47.14	72.71	81.1	52.08	37.61	21.13	91.92	25.29	52.08	32.71	51.82	69.81	95.33	62	64.69	50.52	5.2	28.44	19.72	78.43	55.95	61.97	14.49
PRKAR1A	1059.90	954.49	1.11	0.42	1021.41	1912.41	1183.42	695.35	1020.81	1352.38	1335.01	997.05	724.41	367.94	815	1293.63	858.87	1438.68	899.95	1250.05	1395.17	845.45	1001.11	899.15	374.04	1520.91	881.81	755.33	877.11	593.03	746.22	745.09	820.25	1724.21	926	767.05	724.72
PTEN	449.97	413.70	1.09	0.61	821.34	611.97	300.06	257.33	251.08	539.86	758.53	435.49	479.82	147.17	160.74	636.21	450.32	648.82	452.76	390.62	800.99	361.64	491.88	380.31	376.29	260.92	178.75	455.48	195.38	162.53	309.87	320.71	424.02	759.91	511.57	489.7	265.16
PTGS2	228.68	129.65	1.76	0.03	616	409.94	114.98	208.06	128.08	133.59	331.23	356.7	175.9	8.18	100.87	160.57	246.97	145.39	54.33	92.37	128.03	150.15	44.37	39.12	27.04	264.36	242.3	44.28	181.73	85.66	271.83	28.8	148.81	150.32	109.45	248.91	18.33
PTHLH	554.19	321.38	1.72	0.29	3127.41	304.02	72.91	657.02	442.56	42.09	364.09	577.32	612	241.2	155.08	54.53	39.93	1228.19	12.54	705.47	91.92	252.4	163.96	160.33	173.5	176.24	79.44	29.1	39.66	494.19	198.99	690.38	1521.27	172.3	287.14	225.54	6.39
PTK7	363.62	216.26	1.68	0.03	831.87	213.8	659.02	246.38	275.18	217.77	821.74	246.4	314.33	179.88	275.28	81.8	91.54	318.98	84.98	263.83	134.59	287.92	271.98	150.07	105.9	219.73	583.9	246.72	188.56	151.55	256.22	221.73	385.47	110.51	201.06	229.61	36.66
PTPN11	335.49	242.88	1.38	0.03	663.39	392.29	210.32	240.91	173.73	360.51	611.88	348.11	301.84	102.2	254.07	366.58	273.39	308.13	129.56	237.57	384.08	251.86	185.18	243.07	193.78	283.81	202.58	173.33	227.57	193.28	239.96	187.17	203.49	416.5	269.93	263.14	233.19

PTPRG	105.80	36.84	2.87	0.00	252.72	72.57	50.48	125.93	26.63	60.39	298.36	34.38	84.31	24.53	42.42	196.92	22.73	43.4	25.08	20.23	72.22	28.52	81.01	61.57	63.09	41.2	23.83	10.12	12.68	15.37	11.06	26.64	66.34	46.34	54.11	33.53	14.49
RAD54L	202.65	127.98	1.58	0.13	737.1	49.04	229.95	114.98	226.99	120.78	364.09	127.5	156.12	126.73	145.18	33.33	156.05	71.61	58.51	59.17	52.52	96.87	50.15	39.12	159.98	75.53	341.6	360.58	157.02	213.05	184.69	53.63	117.43	94.47	116.83	137.16	91.66
RAF1	271.85	252.25	1.08	0.54	173.74	390.33	294.45	169.73	361.41	384.3	192.16	315.16	197.76	114.47	259.72	408.99	280.15	321.15	345.49	164.82	397.21	213.11	239.19	261.67	182.51	256.35	198.61	287.2	274.38	208.66	246.14	164.14	172.12	446.8	290.83	200.15	146.65
RARA	178.23	88.98	2.00	0.01	568.62	192.22	129	147.83	81.16	183	300.88	123.2	103.04	77.68	59.39	172.69	74.95	88.97	47.37	92.67	114.9	78.57	100.3	76.96	87.88	109.86	190.66	45.55	56.57	83.46	87.47	45.35	132.67	116.45	85.47	102.61	49.88
RB1	299.12	218.88	1.37	0.05	694.98	203.99	190.69	355.89	190.21	382.47	445	363.87	249.8	147.17	165.45	199.95	331.14	266.9	281.41	169.95	177.27	302.98	231.47	161.62	171.25	199.13	186.69	183.46	229.52	184.5	159.65	90.35	176.6	295.29	278.54	311.9	206.76
REL	72.65	68.17	1.07	0.67	147.42	133.38	72.91	49.28	74.82	73.2	55.63	73.06	65.57	8.18	75.89	42.41	72.49	73.78	62.69	47.09	98.48	37.67	46.29	77.6	78.86	61.8	39.72	126.52	84.2	57.11	84.54	68.75	87.85	82.59	34.43	69.09	40.07
RET	98.74	19.74	5.00	0.00	410.67	54.92	78.52	82.13	5.07	80.52	197.22	81.66	73.9	8.18	36.3	75.74	17.2	19.53	15.32	8.15	39.39	1.61	15.43	12.19	24.79	28.61	67.53	11.39	3.25	13.18	5.2	3.6	23.31	14.26	39.35	40.64	10.66
RRM1	361.70	249.27	1.45	0.02	294.84	196.14	199.11	553	341.12	195.81	480.4	403.98	261.25	678.64	518.03	218.13	259.26	282.09	211.75	213.12	305.3	161.45	119.59	420.08	232.09	258.64	337.63	172.07	217.82	208.66	202.57	242.61	173.01	311.93	553.38	189.99	161.57
S100A4	712.04	629.93	1.13	0.56	779.22	1425.97	538.43	695.35	323.36	1407.35	482.93	477.04	613.04	163.53	399.25	1239.09	798.05	642.31	420.72	464.28	1309.82	865.36	908.53	952.39	414.6	437.16	349.55	531.39	360.53	149.35	510.16	830.4	466.15	1611.33	576.75	416.55	213.15
SERPINE1	1084.87	885.82	1.22	0.62	1189.89	3395.26	243.98	804.86	180.07	527.04	2007.58	1128.84	2782.12	28.62	197.03	533.2	267.86	3890.72	66.87	3538.22	219.95	466.05	935.53	1082.58	24.79	1594.15	301.88	44.28	289.99	129.59	153.15	1315.97	2448.19	319.65	269.31	479.54	763.94
SFPQ	1801.29	1772.21	1.02	0.91	1553.17	1643.69	1895.72	1012.91	4153	1526.23	2070.79	1532.82	1384.29	1017.95	1907.16	1917.72	2306.92	1401.79	1432.13	1256.69	1795.67	1563.36	1375.33	1179.42	2122.57	1290.89	1406.14	3213.63	2772.44	2079.98	2498.15	1750.79	1416.38	2248.84	1608.5	1497.53	999.26
SIAH1	145.80	82.07	1.78	0.02	442.26	123.57	143.02	87.6	158.51	115.29	199.75	117.47	71.82	143.09	59.39	87.86	64.51	108.5	37.61	54.03	111.61	74.8	69.44	28.22	83.37	101.85	123.14	98.69	54.94	208.66	78.36	57.23	134.47	70.7	55.95	52.83	54.57
SOD1	149.07	82.19	1.81	0.02	426.46	262.83	196.3	153.31	100.18	84.18	113.78	77.36	42.67	65.41	66.46	199.95	72.49	78.12	40.4	45.58	131.31	61.35	102.23	66.7	40.56	178.53	67.53	135.38	35.44	204.26	47.47	55.43	60.96	122.39	63.95	57.91	57.98
SPI1	438.50	248.30	1.77	0.03	752.89	413.87	356.15	618.7	256.15	1277.35	541.09	233.5	247.72	53.15	178.18	333.25	252.5	217	257.73	318.47	498.98	99.02	771.57	339.27	196.03	438.31	202.58	105.01	100.78	61.5	146.64	199.77	266.24	278.06	122.97	159.51	182.46
SPP1	1541.90	1145.18	1.35	0.66	342.22	1096.45	201.91	11968.8	32.97	1727.54	336.28	793.63	1462.36	200.32	276.69	63.62	63.89	184.45	26.47	1476.75	65.66	1907.24	2160.4	806.16	1511.93	1111.22	95.33	35.43	339.73	2257.89	173.96	1004.62	610.48	45.75	1586.98	542.53	8041.84
STAT1	1505.75	1390.34	1.08	0.77	1074.06	1161.18	1329.25	782.95	1689.1	4293.22	1582.8	554.39	2092.05	355.67	2666.53	487.76	1556.17	605.42	1205.05	1682.93	1096.44	809.39	5159.88	854.9	680.48	781.63	675.26	3242.73	712.94	2350.14	1160.14	661.59	2073.48	1276.23	485.75	1038.32	1088.36
STAT3	1574.17	1349.27	1.17	0.24	2237.62	2073.25	1741.48	865.08	1629.5	1460.35	1769.91	1694.7	942.99	682.72	1135.53	2656.93	1349.13	2050.6	2196.95	1484.6	2432.53	990.22	1475.63	1300.63	779.63	1514.05	981.12	1056.45	1395.65	1078.43	1442.38	626.31	1202.13	1926.82	969.04	1003.77	1078.55
SYK	257.72	219.62	1.17	0.35	68.44	223.6	294.45	520.14	360.14	497.76	136.54	326.62	149.88	233.03	109.36	172.69	209.5	290.77	284.2	157.88	311.86	290.61	264.26	122.5	322.22	319.29	55.61	191.05	236.67	349.23	162.25	152.98	118.33	252.51	225.04	203.19	92.08
TAL1	133.21	27.02	4.93	0.01	605.47	43.15	28.04	60.23	5.07	166.53	260.43	106.01	113.45	12.26	85.79	112.09	38.09	17.36	15.32	14.49	65.66	9.69	40.51	18.6	18.03	27.47	39.72	8.86	10.73	13.18	11.06	6.84	26	43.37	76.24	58.93	7.25
TEK	19.53	12.99	1.50	0.22	10.53	66.69	22.43	21.9	10.14	20.13	7.59	5.73	6.24	12.26	11.31	39.38	8.6	6.51	11.14	12.07	16.41	8.61	13.5	24.37	9.01	17.17	51.64	3.8	4.55	2.2	4.23	6.84	7.17	38.03	15.99	7.11	3.84
TERT	83.02	13.99	5.94	0.03	500.17	27.46	30.85	54.75	12.68	54.9	141.59	44.41	34.35	20.44	50.44	24.24	17.82	2.17	4.18	3.32	13.13	5.92	38.58	7.7	11.27	13.73	47.67	5.06	9.43	26.36	3.9	2.52	4.48	4.75	25.82	36.57	9.38
TFDP1	377.12	257.58	1.46	0.02	868.72	294.22	353.34	328.51	225.72	272.67	493.05	366.73	375.74	429.26	278.11	239.34	261.1	256.05	256.33	206.78	246.21	348.73	169.75	216.77	119.42	297.54	432.96	482.04	278.28	327.26	168.1	107.62	187.36	285.19	345.56	263.14	153.04
TFE3	111.61	91.83	1.22	0.25	126.36	156.92	78.52	120.45	46.92	124.44	121.36	124.63	86.39	24.53	104.64	224.19	71.27	171.43	65.48	77.88	216.66	67.27	67.51	118.01	60.84	114.44	111.22	44.28	88.43	50.52	41.29	82.43	72.61	142.6	97.76	107.69	58.83
TFRC	947.18	1155.12	0.82	0.39	1669	490.36	507.58	344.94	1162.84	1096.18	1405.81	866.69	1718.4	617.31	1099.23	387.79	642	527.3	454.16	803.28	554.79	1657	630.76	980.6	1721.49	1514.05	1445.86	923.6	3501.96	902.72	829.46	1922.13	418.64	663.07	1808.95	1839.91	515.83
TGFA	151.17	95.27	1.59	0.06	384.34	88.27	114.98	164.26	58.33	106.14	242.73	270.75	196.72	20.44	79.19	87.86	77.41	290.77	139.31	49.2	29.54	189.97	73.3	118.01	166.74	82.4	35.75	106.28	70.87	96.64	55.6	46.79	82.47	60.6	111.91	97.53	19.61
TGFB1	872.27	594.78	1.47	0.05	1642.68	980.72	1954.61	925.31	825.53	827.17	1036.66	710.54	600.55	388.38	305.45	269.63	211.95	1182.62	553.07	665.62	472.72	616.19	977.97	389.29	252.36	714.11	993.03	708.52	502.93	997.16	357.67	481.25	963.68	485.42	438.4	397.24	129.17

TGFBI	1288.44	1611.68	0.80	0.49	1826.95	1357.32	773.99	2217.46	1036.03	1255.39	1496.83	898.21	2607.26	261.64	881.93	848.28	610.06	2334.87	488.98	4920.49	2176.47	1207.63	4363.24	1495.6	671.47	1452.25	746.76	439.03	3059.5	448.06	519.59	1299.06	5112.42	718.92	589.05	757.91	433.98
TGFBR2	221.24	212.14	1.04	0.89	300.1	319.72	106.56	93.08	103.98	430.05	209.86	121.77	227.94	85.85	98.52	557.44	140.69	203.98	103.09	413.26	722.21	85.57	376.14	310.41	45.07	136.18	83.41	75.91	104.03	21.96	66.33	179.97	419.54	651.78	144.49	76.2	94.64
TGFBR3	254.05	148.95	1.71	0.14	405.4	723.77	126.19	76.65	333.51	144.57	217.45	90.25	115.53	130.82	108.89	575.62	140.07	71.61	23.68	70.64	531.81	25.83	81.01	111.59	81.12	125.88	79.44	99.95	323.47	94.44	94.29	66.95	80.68	771.8	113.14	56.89	83.56
THPO	92.56	16.54	5.60	0.00	405.4	39.23	44.87	120.45	3.8	51.24	199.75	48.71	80.14	4.09	43.37	69.68	19.05	21.7	9.75	7.24	29.54	2.15	5.79	9.62	13.52	22.89	79.44	1.27	2.28	4.39	2.6	3.96	5.38	6.54	62.1	35.56	2.56
TIMP1	1145.01	997.32	1.15	0.70	163.21	3581.6	263.61	1565.91	199.09	4231	1044.24	184.8	612	57.23	213.53	1623.85	434.97	1011.2	278.62	2444.85	1135.84	501.57	2432.38	2120.9	60.84	2260.2	1084.39	188.52	136.87	79.07	372.3	1283.22	609.58	827.65	1044.05	1320.76	1316.01
TIMP2	898.75	680.18	1.32	0.40	552.82	3134.39	858.12	744.63	138.22	1703.75	1024.02	153.28	652.6	77.68	312.52	1432.99	140.07	566.36	217.33	676.79	2107.53	647.41	1190.15	1235.86	36.05	1937.48	810.32	136.64	115.08	79.07	202.57	843.36	955.61	1318.41	507.27	372.86	187.57
TIMP3	1416.64	948.26	1.49	0.35	610.74	5841.19	852.51	1007.44	447.64	2648.03	695.32	200.56	499.59	65.41	632.1	3499.16	161.58	303.79	72.44	1571.54	2892.11	244.32	2079.38	2280.59	38.31	925.82	520.35	69.59	164.5	138.37	145.99	2187.77	1492.58	3841.75	325.27	268.21	189.28
TNF	98.41	47.82	2.06	0.01	289.57	90.23	98.15	109.5	43.12	126.27	73.32	110.31	77.02	16.35	83.43	63.62	78.64	62.93	34.83	14.79	39.39	22.6	40.51	16.03	20.28	42.34	198.61	16.45	38.36	24.16	105.02	37.79	89.64	21.98	44.89	36.57	18.33
TNFRSF10B	232.15	128.96	1.80	0.01	673.92	345.21	260.8	175.21	271.37	161.04	275.6	133.23	129.06	106.29	148.01	106.04	74.95	88.97	64.08	81.81	101.77	111.94	57.87	91.07	103.65	113.3	242.3	220.15	181.73	177.91	193.14	153.34	190.94	156.26	146.95	118.87	37.09
TNFRSF1B	543.69	360.16	1.51	0.03	926.64	796.35	409.43	427.07	599.81	1054.09	720.6	310.86	372.61	49.06	300.26	557.44	344.66	334.17	455.55	474.54	692.66	190.51	686.7	380.31	164.49	617.98	222.44	227.74	173.6	182.3	301.74	245.13	587.17	553.15	203.52	226.56	298.41
TNFSF10	1457.69	1388.41	1.05	0.86	1526.85	400.14	2636.06	317.56	3488.52	898.54	1663.71	1272.1	1776.68	1279.6	1635.65	596.83	1188.17	1347.54	1967.08	1672.36	817.41	465.51	3771.06	546.42	1631.36	669.48	274.08	4734.41	1053.97	687.47	1852.72	572.32	2190.91	1556.66	481.44	591.29	1084.95
TOP1	694.70	614.67	1.13	0.11	747.63	963.07	636.58	602.27	767.2	839.98	644.75	690.49	792.07	302.52	713.65	636.21	876.08	805.05	558.64	480.58	610.59	597.9	488.02	495.75	684.99	567.62	536.24	774.31	751.63	465.64	569.34	668.79	474.22	696.34	724.93	577.07	504.32
TOP2A	232.56	212.85	1.09	0.58	379.08	88.27	221.54	186.16	234.6	111.63	477.87	177.64	303.92	412.91	124.44	72.71	305.95	171.43	174.14	261.72	91.92	171.14	177.46	118.65	180.26	140.76	313.8	202.43	289.99	300.91	254.92	163.42	164.05	129.52	259.48	332.22	265.59
TP53	300.88	167.16	1.80	0.00	547.56	235.37	280.43	169.73	603.61	239.73	402.02	210.58	155.08	241.2	246.53	278.72	90.93	230.01	266.09	79.09	226.51	131.31	131.17	114.8	159.98	127.03	111.22	123.99	59.82	70.28	467.57	209.13	118.33	182.4	375.69	141.22	93.79
TPR	182.80	214.08	0.85	0.16	126.36	156.92	117.78	76.65	300.54	210.45	192.16	209.15	195.67	196.23	223.9	187.83	310.25	232.18	193.64	200.74	275.75	264.78	185.18	132.12	198.29	124.74	170.8	222.68	329.65	129.59	203.22	152.98	164.05	301.23	215.82	275.33	212.73
TYMS	388.45	384.14	1.01	0.95	673.92	413.87	277.63	361.36	322.1	278.16	690.26	320.89	498.55	331.14	366.25	127.24	1061	219.17	286.98	267.16	252.77	201.81	339.49	166.75	567.82	215.15	504.46	584.53	573.15	417.31	327.43	177.09	309.27	251.92	475.91	402.32	465.53
TYRO3	251.57	99.33	2.53	0.01	942.43	125.53	143.02	175.21	72.28	168.36	366.62	312.29	236.27	81.76	167.81	227.22	53.45	143.22	112.84	37.43	131.31	129.16	69.44	78.24	81.12	86.97	139.02	118.93	73.8	94.44	68.61	77.39	41.24	118.24	193.68	156.46	81
WEE1	123.78	14.96	8.27	0.00	605.47	54.92	44.87	98.55	12.68	107.97	202.27	80.22	73.9	24.53	89.09	90.89	41.16	13.02	12.54	3.92	9.85	1.61	19.29	10.26	13.52	17.17	27.8	5.06	3.9	13.18	4.23	2.16	2.69	6.54	50.42	47.75	8.1
WFDC2	225.16	186.98	1.20	0.67	515.97	290.29	70.11	104.03	123	550.84	318.58	38.68	344.51	49.06	78.72	218.13	646.31	32.55	54.33	23.55	833.82	3.23	156.24	30.78	27.04	29.75	711.01	5.06	28.93	101.03	24.71	6.48	50.2	604.84	30.13	515.09	11.51
WNT1	91.37	10.72	8.53	0.01	431.73	29.42	11.22	60.23	7.61	100.65	245.26	67.33	57.25	20.44	47.14	18.18	21.5	6.51	6.97	1.81	3.28	2.15	15.43	7.7	9.01	13.73	23.83	1.27	1.3	13.18	3.25	1.44	0.9	4.16	43.66	42.67	1.28
WNT10B	90.50	13.32	6.79	0.00	379.08	41.19	42.06	49.28	3.8	109.8	209.86	83.09	71.82	20.44	45.25	30.3	19.66	4.34	12.54	2.11	9.85	2.69	27	4.49	15.77	10.3	15.89	1.27	3.9	4.39	39.02	4.68	10.76	14.26	38.12	31.49	7.25
WT1	107.73	17.51	6.15	0.01	484.38	64.73	39.26	38.33	2.54	84.18	275.6	110.31	89.51	4.09	51.38	48.47	11.67	17.36	11.14	14.49	26.26	2.15	38.58	14.75	9.01	30.9	39.72	6.33	3.58	10.98	3.25	10.44	8.96	13.07	54.11	37.59	3.41
XPC	143.66	127.32	1.13	0.50	73.71	294.22	204.72	120.45	289.13	197.64	111.25	81.66	58.29	106.29	74.48	112.09	107.51	91.14	136.53	86.64	246.21	86.11	109.95	96.84	101.4	146.48	143	165.74	99.15	136.18	145.34	107.62	135.36	286.38	108.83	69.09	68.21
XRCC5	1888.88	1581.84	1.19	0.09	1769.04	1686.84	2016.31	1784.92	3090.34	1937.99	1888.74	1700.43	1440.5	1001.6	2695.75	1654.15	1739.86	1716.43	1503.17	1256.69	1812.09	1703.28	1440.91	1262.15	2075.25	1642.22	1171.78	2693.63	2161.9	1370.55	1503.51	830.04	1006.71	2220.33	1906.71	1172.42	1029.1
YES1	520.33	478.82	1.09	0.59	1221.48	598.24	319.69	487.29	285.32	468.48	670.04	587.34	493.35	122.65	469.01	521.09	919.08	562.02	502.92	384.58	594.18	429.45	385.79	439.32	549.79	344.47	242.3	377.03	663.2	287.73	343.36	224.61	380.99	598.31	635.16	553.7	637.33
YY1	405.28	442.53	0.92	0.51	215.86	500.17	443.08	383.26	634.05	424.56	169.41	362.43	383.02	584.61	305.45	457.47	452.17	494.75	395.65	300.96	410.35	375.64	335.63	328.36	1025.23	431.44	456.8	641.46	582.58	470.03	322.88	379.75	337.96	501.46	372	420.61	257.49

Supplementary Table 2: Loci information of somatic mutations (N=36)

Gene	Frequency (Total)	Frequency (PFS ≥ 4 months)	Frequency (PFS < 4 months)	PFS ≥ 4 months											PFS < 4 months																								
TP53	61%	55%	64%	R175H	E294*	E221*		V274A	Y236*				I232F		E294K	D186N.A161A.P98L			R282Q	R196*	Y220C	Y220C	P278L	E204*		T304A.E286K		S183*	G245S	R280*		Y236C.R202H	S166A			R273H	A78V		
CDKN2A	8%	9%	8%	A76fs*70																			P114L							W110*									
MLH1	8%	18%	4%		V384D									V384D																		V384D							
STK11	3%	9%	0%					P281L																															
PIK3CA	3%	0%	4%																		H1047R																		
PTEN	3%	0%	4%																																	R130Q			
FLT3	8%	18%	4%					Y842C					Y842C			Y842C																							
EGFR	8%	9%	8%											V765M					G810D			C775Y																	
KIT	3%	0%	4%																															D820E					
APC	6%	9%	4%							Q886*																											S1355P		
CTNNB1	6%	9%	4%					S37F														A39T																	
IDH2	3%	0%	4%																													R172K							
IHD1	3%	9%	0%					R132C																															

PFS: progression free survival

Supplementary Table 3: Gene list and platforms for biomarker analysis

Data type	DNA mutation	Gene expression
Platform	AmpliSeq™ Cancer Hotspot Panel v2	nCounter GX Human Cancer Reference Kit
No. of genes	50 genes (2800 hot spots)	230 genes
Gene lists	*SMARCB1*	*ABCB1*
	RB1	*ABL1*
	TP53	*AKT1*
	ERBB4	*AKT2*
	FBXW7	*APC*
	BRAF	*AR*
	KIT	*AREG*
	GNAS	*ATM*
	HRAS	*BCL2*
	EGFR	*BCL2A1*
	PDGFRA	*BCL2L1*
	PIK3CA	*BCL3*
	CDKN2A	*BCL6*
	ERBB2	*BCR*
	ABL1	*BIRC2*
	JAK2	*BIRC5*
	KRAS	*BLM*
	NRAS	*BMI1*
	NOTCH1	*BRAF*
	ATM	*BRCA1*
	FGFR1	*BRCA2*
	STK11	*CASP10*
	PTPN11	*CASP2*
	APC	*CAV1*
	SMAD4	*CCNA2*
	PTEN	*CCND1*

SMO
CTNNB1
RET
IDH2
SRC
EZH2
VHL
MPL
NPM1
FLT3
FGFR3
CDH1
KDR
HNF1A
MLH1
ALK
IDH1
GNAQ
AKT1
JAK3
FGFR2
GNA11
MET
CSF1R

CCND2
CCND3
CCNE1
CD34
CD44
CDC2
CDC25B
CDC25C
CDH1
CDH11
CDK2
CDK4
CDK6
CDKN1A
CDKN2A
CDKN2B
CDKN2C
CEBPA
CHEK1
COL1A1
CSF1R
CSF3
CSF3R
CSK
CTGF
CTNNB1
CXCL9
CYP1A1
DAP3
DAPK1
DEK

DLC1
E2F1
E2F3
EGF
EGFR
EGR1
EPS8
ERBB2
ERBB3
ERBB4
ERCC2
ERCC4
ESR1
ETS1
ETS2
ETV1
ETV6
FANCG
FAS
FAT1
FGF1
FGF2
FGFR1
FGFR2
FGFR3
FGFR4
FGR
FLT1
FLT3
FOLR1
FOS

FOSL2
FRZB
FYN
GADD45A
GAS1
GATA1
GNAS
GRB7
HCK
HDAC1
HIF1A
HMMR
HRAS
HSP90AB1
IFNGR1
IGF1
IGFBP2
IGFBP3
IGFBP6
IL1A
IL1B
IL4
IL6
IL8
IRF1
ITGB1
JUN
JUNB
KDR
KIT
KRAS

L1CAM
LAMB1
LCK
LIF
LMO1
LMO2
LYN
MAP3K8
MAPK10
MCL1
MET
MLH1
MLL
MMP1
MMP14
MMP2
MMP3
MMP9
MPL
MSH2
MSH6
MST1R
MTA1
MUC1
MYB
MYBL2
MYC
MYCL1
MYCN
NF1
NGFR

NOTCH1
NPM1
NQO1
NRAS
NTRK1
NTRK2
NTRK3
NUMA1
OGG1
PCNA
PCTK1
PDGFA
PDGFRA
PDGFRB
PIK3CA
PIM1
PLA2G2A
PLAT
PLAUR
PLG
PML
PPARG
PRKAR1A
PTEN
PTGS2
PTHLH
PTK7
PTPN11
PTPRG
RAD54L
RAF1

RARA
RB1
REL
RET
RRM1
S100A4
SERPINE1
SFPQ
SIAH1
SOD1
SPI1
SPP1
STAT1
STAT3
SYK
TAL1
TEK
TERT
TFDP1
TFE3
TFRC
TGFA
TGFB1
TGFBI
TGFBR2
TGFBR3
THPO
TIMP1
TIMP2
TIMP3
TNF

TNFRSF10B
TNFRSF1B
TNFSF10
TOP1
TOP2A
TP53
TPR
TYMS
TYRO3
WEE1
WFDC2
WNT1
WNT10B
WT1
XPC
XRCC5
YES1
YY1
